# Investigating the Neuroprotective Effects of Turmeric Extract: Structural Interactions of *β*-Amyloid Peptide with Single Curcuminoids

**DOI:** 10.1038/srep38846

**Published:** 2016-12-22

**Authors:** Rosario Randino, Manuela Grimaldi, Marco Persico, Augusta De Santis, Elena Cini, Walter Cabri, Antonella Riva, Gerardino D’Errico, Caterina Fattorusso, Anna Maria D’Ursi, Manuela Rodriquez

**Affiliations:** 1 Department of Pharmacy, University of Salerno, Via Giovanni Paolo II, 132, 84084-Fisciano-Italy; 2 Department of Pharmacy, University of Naples “Federico II”, Via D. Montesano, 49, 80131-Naples-Italy; 3 Department of Chemical Sciences, University of Naples “Federico II”, Via Cinthia, 80126-Naples-Italy; 4 Department of Biotechnology, Chemistry and Pharmacy, University of Siena, Via Aldo Moro, 2, 53100-Siena-Italy; 5 R&D Department, Indena, Viale Ortles, 12, 20139-Milan-Italy; 6 Innovation & Development Fresenius-Kabi, Piazza Maestri del Lavoro, 7, 20063-Cernusco sul Naviglio Milan-Italy

## Abstract

A broad biophysical analysis was performed to investigate the molecular basis of the neuroprotective action of *Curcuma longa* extracts in Alzheimer’s disease. By combining circular dichroism and electron paramagnetic resonance experiments with molecular modeling calculations, the minor components of *Curcuma longa* extracts, such as demethoxycurcumin (**2**, DMC), bisdemethoxycurcumin (**3**, BDMC) and cyclocurcumin (**4**, CYC), were analyzed in a membrane environment mimicking the phospholipid bilayer. Our study provides the first evidence on the relative role of single curcuminoids interacting with A*β*-peptide. When the CYC and curcumin metabolite tetrahydrocurcumin (**5**, THC) were inserted into an anionic lipid solution, a significant modification of the A*β* CD curves was detected. These data were implemented by EPR experiments, demonstrating that CYC reaches the inner part of the bilayer, while the other curcuminoids are localized close to the membrane interface. Computational studies provided a model for the curcuminoid-A*β* interaction, highlighting the importance of a constrained “semi-folded” conformation to interact with A*β* analogously to the pattern observed in α-helical coiled-coil peptide structures. This combined approach led to a better understanding of the intriguing *in vitro* and *in vivo* activity of curcuminoids as anti-Alzheimer agents, paving a new path for the rational design of optimized druggable analogues.

Alzheimer’s disease (AD) is a progressive neurodegenerative disorder and the most common cause of dementia in adults. The mechanisms underlying the disease are currently in question, but the deposition of senile plaques composed by fibrillar aggregates of amyloid *β* peptide (A*β*) is a characteristic hallmark of the pathology and is considered fundamental in disease pathogenesis^1^,^2^,^3^,^4^. The main components of the plaques are the peptides A*β*(1–40) and A*β*(1–42), which occur as fibrillar aggregates in equilibrium with soluble oligomers and random coil or *α*-helical monomers^5^,^6^,^7^,^8^. *Curcuma longa* is a Southeast Asian plant, having an orange rhizome with a strongly aromatic smell. The rhizome is normally dried and then crushed for gastronomic use as a spice turmeric powder and also as a colorant in food (additive E100) and textile industry^9^. The mixture (MIX) of curcuminoids, obtained as an extract from the turmeric powder of *Curcuma longa* (Fig. 1A), is constituted by the most abundant curcumin (**1**, CUR), demethoxycurcumin (**2**, DMC), bisdemethoxycurcumin (**3**, BDMC) and cyclocurcumin (**4**, CYC), which is present in small amounts. They are provided with important pharmacological activities, such as antioxidant^10^, antiangiogenic^11^, antiinflammatory^12^, antiviral^13^ and anticancer^14^.

Worldwide comparison of incidence studies promoted by the World Health Organization shows that the incidence rates of AD reported in the populations of South East Asia are lower than in Europe and North America. People living in rural compared to urban settings have lower rates of AD: United States ranks 2, Italy 21 and India 165 among 172 countries^15^,^16^, suggesting the existence of undefined protective or risk factors^17^.

The lower incidence rates of AD in the populations of South East Asia, the main consumers of turmeric, are associated with regular curcumin consumption, which is efficacious to counteract the neurophysiological damages occurring during the disease.

However, the low bioavailability of turmeric components greatly limits its use in therapy. The daily intake of a considerable amount of turmeric - up to 3.6 g/*die* - is converted into nanomolar concentrations in the plasma. Pharmacokinetic analysis of curcumin in humans showed that in the peripheral circulation, the main metabolites present were curcumin glucuronide and curcumin sulfate^18^,^19^, whereas other reduced metabolites resulting from the metabolic transformation of curcumin were detected in traces. In particular, after oral administration, the analysis of rat plasma concentrations by HPLC revealed the presence of tetrahydrocurcumin (**5**, THC), hexahydrocurcumin (**6**, HHC) and octahydrocurcumin (**7**, OHC) (Fig. 1B)^20^,^21^, essentially curcuminoids resulting from subsequent metabolic reactions of curcumin (**1**, CUR).

One of the strategies used to overcome the low bioavailability of curcumin involves the co-administration of turmeric with piperine, a component of *Piper nigrum* that inhibits the human enzymes CYP3A4 and P-glycoprotein, which are important for the metabolism and transport of xenobiotics and metabolites^22^. Moreover, as the interaction with lipid layers enhances the bioactivity of curcumin^23^, curcuminoids from turmeric rhizome of *Curcuma longa*, thus containing the four curcuminoids as in the MIX, have been commercialized as the highly standardized lecithin-formulated extract Meriva^®^, developed according to the Indena Phytosome^®^ technology. Total curcuminoids absorption was about 29-fold higher for Meriva^®^ than for its corresponding unformulated curcuminoids extract^24^,^25^,^26^.

Considering the implication of A*β* in oligomeric or fibrillar aggregates in the etiology and progress of Alzheimer’s disease, thousands of molecules have been screened to identify new substances that can control the formation of *β* amyloid fibrils, either stabilizing the soluble peptide conformation or dissolving aggregates^27^,^28^,^29^,^30^. Indeed, among the substances tested for preventing or disaggregating *β*-amyloid fibrils, curcumin and several synthetic curcumin derivatives have been shown to bind A*β*, affecting the process of fibril formation^31^,^32^,^33^,^34^,^35^,^36^. In particular, the analysis of curcumin derivatives highlighted the importance of keto-enol tautomerism, proving that the derivatives existing predominantly in the enol-form have higher affinity for A*β* aggregates^37^,^38^,^39^. Among the curcumin derivatives, minor attention has been reserved for the remaining curcuminoids present in turmeric extract and their metabolites, although increasing evidence has led to the hypothesis that the activity of each of these compounds is determinant for the ultimate biological effect of *Curcuma longa* extract. Indeed, i) the turmeric extract containing optimized proportions of the single curcuminoids and integrated with a given proportion of the reduced metabolite THC exhibits enhanced biological activity^40^; ii) the turmeric phytocomplex is therapeutic despite the unfavorable pharmacokinetic properties of curcumin alone; and iii) metabolites resulting from the metabolic transformation of CUR, such as THC, HHC and OHC (Fig. 1B), are themselves biologically active^20^,^21^.

While the structural interaction between curcumin and A*β*-peptide is known^31^,^32^,^33^,^34^,^35^,^36^, studies on the possible structural interactions with A*β*-peptide of other compounds structurally related to curcumin present in turmeric extract or deriving from curcumin metabolism are missing. To overcome this gap, using a combined approach including circular dichroism (CD) and electron paramagnetic resonance (EPR) spectroscopy, we analyzed the mixture extracted from *Curcuma longa* (MIX), all the single components (CUR, DMC, BDMC and CYC, Fig. 1A) and their metabolites (THC, HHC, OHC Fig. 1B) for their ability to interact with the *β* amyloid fragment *Aβ*(25–35). Using an advanced simulation protocol, we acquired data to read all the collected experimental data into molecular level.

Many *β-*amyloid fragments have been studied *in vitro* and *in vivo*, and are currently the focus of biophysical and computational investigations^41^. The choice of *Aβ*(25–35) was based on the evidence that this is the shortest fragment of the *β-*amyloid peptide that forms toxic aggregates, preserving the same activity of the full-length A*β*-peptide^42^,^43^,^44^,^45^,^46^,^47^. Moreover, our previous studies evidenced that, under several different environmental conditions, *Aβ*(25–35) presents the same conformational behavior as the corresponding fragment in the full length Aβ(1–42)^46^,^48^. In this respect, it is reasonable to use *Aβ*(25–35) for testing molecules which may act on the equilibrium monomer-oligomer-fibril of the full length peptide.

Finally, we decided to undertake our biophysical study in the membrane-mimicking environment represented by a liposomal solution of different composition. This choice was dictated by the critical role played by the membrane surface in the *β*-amyloid toxicity, associated with the curcuminoids’ aptitude for interaction with membranes, enhancing their biological effects^23^,^49^,^50^,^51^.

Our study provides the first evidence regarding the relative role of the individual curcuminoid interacting with A*β*-peptide in membrane-mimicking environments. Among the examined curcuminoids, only CYC and THC can modify the A*β* conformation in the liposome system. Computational studies provide a molecular model for curcuminoid-A*β* interaction, suggesting that the constrained “semi-folded” conformation of CYC is suitable for interaction with A*β,* reproducing the pattern observed in α-helical coiled-coil peptide structures.

## Results

### Analysis of curcuminoids

**Figure 1:**
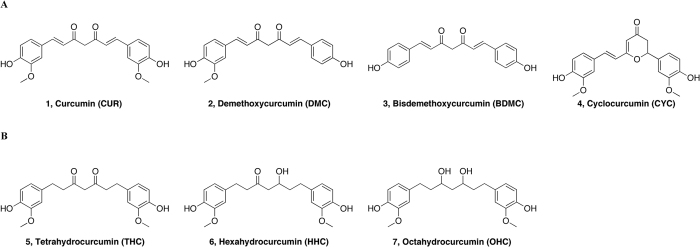
(**A**) Structures of natural curcumin derivatives, curcuminoids, (1–4). (**B**) Structures of main curcumin metabolites (5–7).

**Figure 2:**
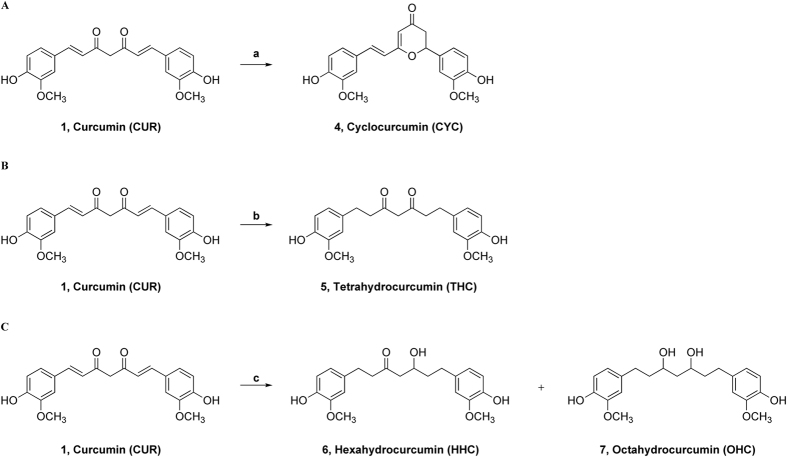
(**A**) Synthesis of cyclocurcumin, CYC (4); reagents and conditions: a) TFA, MW, 100 °C, 4 min, 10%. (**B**) Synthesis of THC (5); reagents and conditions: b) H_2_ (1 atm), Pt/C, MeOH, r.t., 1 h, 77%. (**C**) Synthesis of HHC (6) and OHC (7); reagents and conditions: c) H_2_ (6.8 atm), Pt/C, MeOH, r.t., 13 h; 6, HHC (37%); **7**, OHC (55%).

We analyzed the curcuminoids present in Meriva^®^ using a different HPLC elution system (chromatograms obtained are shown in Supporting Information in Figure 1SI and 2SI). The chromatogram obtained under these new conditions showed the presence of CUR (retention time (rt) = 20.44 min), DMC (rt = 19.97 min), BDMC (rt = 19.50 min) and another component with the rt of 13.90 minutes. We therefore hypothesized that the new peak could correspond to cyclocurcumin. To validate our hypothesis, we embarked on the synthesis of cyclocurcumin to fully characterize the new entity via HRMS and NMR and then to evaluate its rt by HPLC analysis. The chromatogram obtained (Figure 2SI) was compared with the previous one (Figure 1SI), showing that the two had equal retention times. A quantitative analysis of curcuminoids present in the MIX, calculated as the percentage of the area under the curve, was performed, with the following results: 83% CUR, 11% DMC, 4% BDMC, and 2% CYC.

The three most abundant natural curcuminoids present in extracts **1**, **2**, and **3** were obtained by flash chromatography on silica gel using the eluent mixture DCM/MeOH (97:3). Each compound was subsequently characterized via HRMS and ^1^H-^13^C NMR. The purity of each isolated compound was assessed by HPLC analysis (see Supporting Information).

### Synthesis of curcuminoids

Since the quantitative analysis showed 2% cyclocurcumin in the mixture, we opted to synthesize this curcuminoid. The general reaction scheme is depicted in Fig. 2A.

Several attempts were made to obtain CYC (see Table SI1 in Supporting Information). We first reproduced the acid-catalyzed cyclization of curcumin as reported by Kiuchi and coworkers^52^ using benzene and TFA (trifluoroacetic acid) for 65 h in the dark. The reported reaction yield is 20%; nevertheless, in our hands, the same reaction never provided a yield higher than 5%, probably because of the difficulties shown in the purification process. We attempted to modify the acidity of the reaction medium by replacing the TFA with TfOH (trifluoromethanesulfonic acid) (Table 1SI); however, the yield did not improve. A reduction of both the reaction time and the amount of by-products was achieved using microwave-assisted synthesis in benzene. The best result was obtained through a solvent-free reaction, performing just one 4 min MW run using TFA at 100 °C. Under these conditions, the reaction yield was 10%, barely sufficient to accumulate the desired quantities despite being completed in only a few minutes.

Among the metabolites formed after the administration of curcumin, the most interesting are THC, HHC and OHC, which are obtained after the reduction *in vivo* of curcumin. In all these compounds, the extended conjugation is lost, and in the case of HHC and OHC, the keto-enol tautomerization is lost as well. This behavior was very interesting as a way to assess the actual importance of tautomerism in the pharmacodynamics of curcumin.

The synthesis of these three metabolites was conducted through catalytic hydrogenation, suitably varying the reaction time and H_2_ pressure^53^. THC was obtained in 77% yield using H_2_ (1 atm), Pt/C, and MeOH for 1 h at room temperature (Fig. 2B).

HHC and OHC were obtained in a 40:60 mixture using higher hydrogen pressures and longer reaction times in a Parr^®^ apparatus (6.8 atm H_2_, Pt/C, MeOH at room temperature for 13 h (Fig. 2C). HHC and OHC were obtained with yields of 37% and 55%, respectively.

### Circular dichroism analysis

Biological and biophysical experiments show that curcumin can interact with A*β*-peptide in soluble or fibrillar form^31^,^32^,^33^,^34^,^35^,^36^. The correlation between the pathological role of A*β*-peptide and its interaction with the neuronal membrane is well established^23^,^49^,^50^,^51^,^54^. The phytosomal formulation of curcuminoids (Meriva^®^) is proven to enhance the biological action of curcumin in terms of plasmatic availability^24^,^26^ and penetration in tissues characterized by amyloid plaque deposition^55^.

**Figure 3:**
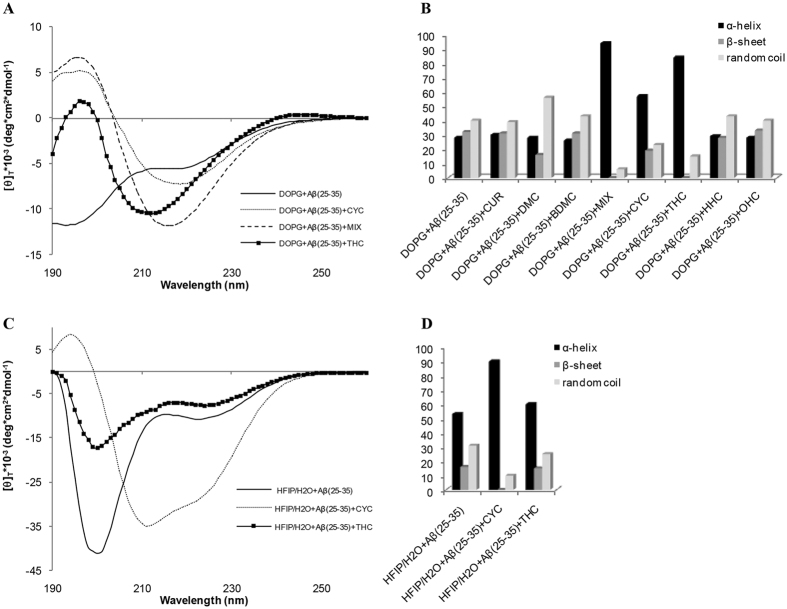
(**A**) CD spectra of A*β*(25–35) in DOPG vesicles in the absence and the presence of ligands MIX, (**B**) Secondary structure quantification of A*β*(25–35) in DOPG vesicles in the presence of curcuminoid compounds. (**C**) CD spectra of A*β*(25–35) in HFIP/water mixture in the absence and the presence of ligands MIX, (**D**) Secondary structure quantification of A*β*(25–35) in HFIP/water mixture in the presence of curcuminoid compounds.

To test the hypothesis that all these actions might share a common biophysical mechanism related to the interaction of A*β* with curcumin at the biomembrane, we analyzed A*β*(25–35) in liposomal solution by CD spectroscopy (Fig. 3A,B), focusing on the variation of its conformation in the presence of the individual curcuminoids shown in Fig. 1. We chose as the liposome systems small unilamellar vesicles (SUV) composed of 1,2-dioleoyl-sn-glycero-3-phosphocholine (DOPC) and/or 1,2-dioleoyl-*sn*-glycero-3-phospho-*rac*-(1-glycerol) (DOPG). Varying the lipid constituents can suitably modulate the liposome composition. The constituents can differ in the length and number of the methylene chains, as well as the polarity and charge of the polar heads. Given the importance of the superficial charge in driving the behavior of the molecules on the membrane surface, we decided to record CD spectra in three different liposome solutions distinguished by different superficial charges. DOPC is characterized by a zwitterionic membrane surface, DOPG by a negatively charged surface, and DOPC/DOPG (90/10 molar ratio) by a zwitterionic surface doped with a small amount of negative charge.

A*β*(25–35) was obtained in complete monomeric form, according to the defibrillating procedure described by Zagorski^56^. CD spectra of monomeric A*β*(25–35) (100 μM solution) were recorded in DOPC, DOPG and DOPC/DOPG liposomal solution (200 μM solution) (see Supporting Information). The qualitative and quantitative analysis of CD curves (DICHROWEB online server)^57^ indicates that A*β*(25–35), in all the examined solutions, exists in an equilibrium of random coil (slightly predominant), α-helix, and β-sheet conformations (Fig. 3B). CD spectra recorded in DOPC and DOPC/DOPG liposomal solutions after the addition of curcuminoids show no significant modification of the A*β*(25–35) conformation in DOPC and DOPC/DOPG liposome solution. Conversely, A*β*(25–35) in DOPG solution, containing CYC and THC, undergoes a marked increase in α-helix structures, as shown by the quantitative evaluation of the CD spectra reported in Fig. 3A.

To estimate the proportions in which these conformational modifications were attributable to direct A*β*(25–35)/curcuminoid interaction or were mediated by the phospholipid bilayer, we recorded CD spectra in mixture of water/organic solvents. An exhaustive conformational analysis of A*β*(25–35) in a hexafluoro-2-propanol (HFIP)/water mixture containing different proportions of organic solvent and water was previously conducted in our lab^45^. This study proved the ability of HFIP to stabilize monomer helical *Aβ*(25–35) conformations. Thus, proceeding from our previous experience, we recorded CD spectra of A*β*(25–35) in HFIP/water 80/20 v:v in the absence and in the presence of CYC and THC. Analysis of the CD spectra shows that, even in these conditions, CYC modifies the conformational behavior of A*β*(25–35) (Fig. 3C,D), whereas THC induces only modest modifications of the CD spectra. These results indicate that the ability of CYC to destabilize the conformational arrangement of A*β*(25–35) is due to a direct CYC-peptide interaction instead of a mediated mechanism involving the lipid membrane, as in the case of THC.

### EPR experiments

**Figure 4:**
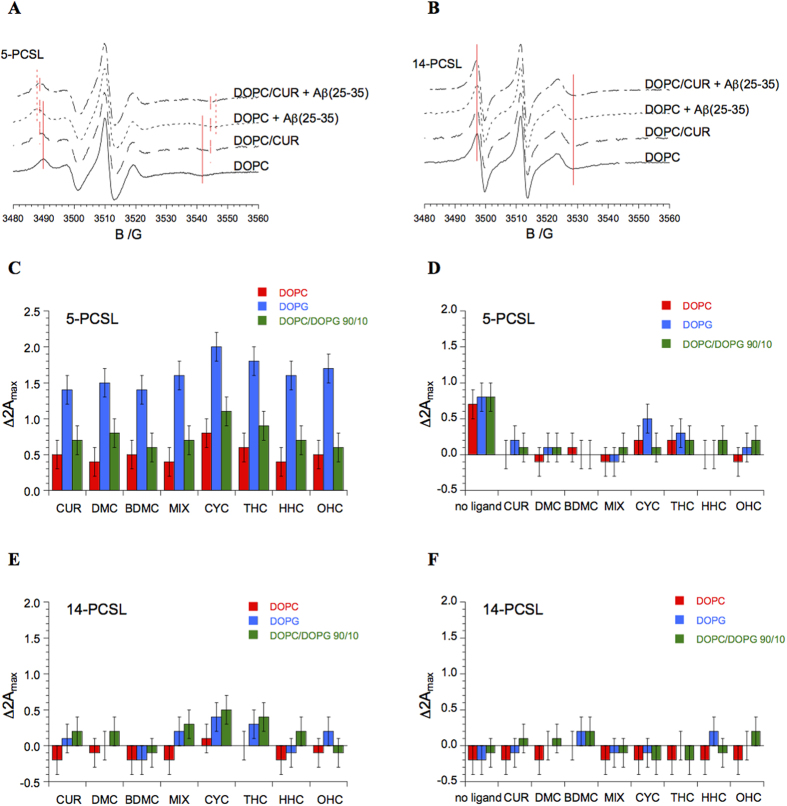
EPR spectra of 5-PCSL (**A**) and 14-PCSL (**B**) in lipid bilayers composed of DOPC or DOPC and CUR, in the absence and in the presence of A*β*(25–35). Red lines are guides for the eye, highlighting the shifts of the low-field maximum and high-field minimum. Increase in outer hyperfine splitting of 5-PCSL and 14-PCSL in DOPC, DOPG and DOPC/DOPG 90/10 bilayers upon inclusion of ligands (CUR, DMC, BDMC, MIX, CYC, THC, HHC and OHC) in the lipid formulation (panels C,E) and upon addition of A*β*(25/35) to the system (panels D,F).

The CD experiments suggest that a significant perturbation of A*β*(25–35) conformational preferences could be induced by the peptide interaction with CYC and THC. These interactions are modulated by the presence of lipid membranes in the case of THC and MIX, whereas CYC can modify the behavior of A*β*(25–35) directly. To investigate this effect more deeply, we performed EPR analysis using experimental conditions consistent with the ones adopted for CD analysis. EPR spectroscopy requires the presence of unpaired electrons in the system to be investigated. To study microstructured systems, specific components can be selectively labeled with radical moieties, thus making it possible to obtain detailed information on the specific site where the label is positioned. In this work, we have used phospholipids specifically spin-labeled at different positions along the acyl chain. 5-PCSL is labeled very close to the hydrophilic head-group, thus allowing observation of the behavior of the bilayer region just below the interface. In contrast, 14-PCSL allows analysis of the properties of the inner part of the bilayer hydrophobic core^58^. Using these two spin-labels, we focused on two different aspects of the systems under consideration in this work. First, we studied the interaction of curcumin and its analogues with the lipid membranes formed by DOPC, DOPG and DOPC/DOPG (90/10 molar ratio); second, we analyzed the effects of these ligands on the interaction of the A*β*(25–35) peptide with the lipid bilayers. The 5-PCSL spectrum in DOPC bilayers (Fig. 4A, solid line) shows a clearly defined axially anisotropic behavior, as detectable by the splitting of the low- and high-field lines, indicating an ordered organization of the outer segments of the lipid acyl chains. The 14-PCSL spectrum, registered in the same system, shows an almost isotropic three-line lineshape (Fig. 4B, solid line).

Very similar results were obtained for the DOPG and DOPC/DOPG (90/10 molar ratio) bilayers (spectra not shown). This evidence indicates, for all the considered lipid systems, an increase flexibility in terms of the segmental chain mobility moving inward from the polar head-groups to the inner hydrophobic core, as expected for membranes in the liquid-disordered (L_d_) crystalline state^59^. To quantitatively analyze the spectra, the values of the outer hyperfine splitting, 2A_max_, were determined by measuring the difference between the low-field maximum and the high-field minimum, using a home-made MATLAB-based software routine. In general, 2A_max_ is dependent on both the amplitude (i.e., order) and the rate of chain rotational motion and is therefore a useful parameter for characterizing chain dynamics, as determined by local ordering, in phospholipid membranes^60^. It is a sensitive means for detecting and quantifying lipid bilayer interactions with guest molecules, as previously demonstrated^61^. The 2A_max_ value is much higher for 5-PCSL than for 14-PCSL (approximately 51 G vs. 32 G) and is quite insensitive to the lipid charge (for 2A_max_ values for 5-PCSL and 14-PCSL in DOPC, DOPG and DOPC/DOPG (90/10 molar ratio) bilayers (Table 2SI in Supporting Information). In the presence of curcumin, the anisotropy of the 5-PCSL spectrum in DOPC increases slightly but significantly, as shown by Fig. 4A, dashed line. The anisotropy increase indicates a motional restriction of the spin-labeled chain and is usually related to the presence of guest molecules^61^. In contrast, the 14-PCSL spectrum remains almost unaffected (Fig. 4B). These results indicate that curcumin interacts with the bilayer, positioning close to the head-group region with a limited but still detectable intercalation among the outer segments of the lipid acyl chains. This conclusion is in reasonable agreement with the “surface association” of curcumin proposed by Sun^62^, while confirming a certain degree of internalization as found by Barry^23^,^54^.

Qualitatively similar effects were observed for DOPG and DOPC/DOPG (90/10 molar ratio) bilayers, perturbation being slightly higher for the anionic lipid system. Consistently, the 2A_max_ values reported in Table 2SI show an increase for 5-PCSL, while remaining almost constant in the case of 14-PCSL. The analogues of curcumin considered in this work cause variations in the 5-PCSL and 14-PCSL 2A_max_ values that are qualitatively similar to the ones observed for CUR (see Table 2SI).

To easily compare the effects of the various ligands on the bilayer structure, Fig. 4 (panels C–E) reports the 2A_max_ variation upon ligand inclusion in all the considered systems for both spin-labels. In the case of 5-PCSL, the variation is positive in all cases and higher for the DOPG bilayers.

In the case of 14-PCSL, the 2A_max_ variations are almost negligible. Thus, it can be concluded that all CUR derivatives present a marked tendency to interact with the bilayers, shallowly penetrating between the lipid acyl chains. The differences between the various ligands are not dramatic, even though CYC seems more effective in perturbing the membrane inner layer. Indeed, for this ligand, even the 14-PCSL 2A_max_ in anionic membranes is slightly but significantly perturbed. Then, we turn our attention to the interaction of the A*β* peptide with lipid membranes, analyzing the extent to which it is affected by the presence of curcumin and its analogues.

The addition of A*β*(25–35) significantly increases the anisotropy of the 5-PCSL spectrum in DOPC (see Fig. 4A, dotted line). Accordingly, the peptide causes a clear 2A_max_ increase (Table 2SI), i.e., the mobility of the spin-labeled chains decreases through the interaction of the peptide with the membrane. In contrast, the addition of A*β*(25–35) to DOPC bilayers does not causes any change in the 14-PCSL spectrum (Fig. 4B). This evidence indicates that A*β*(25–35) is inserted in the bilayer positioning between the outer part of the hydrophobic core and the external hydrophilic layer. A similar solubilization site was already observed in other lipid systems^58^,^63^. When A*β*(25–35) is added to the lipid bilayers in the presence of curcumin or its analogues, the 5-PCSL spectrum is much less affected, and in some cases, no variation is observed (see Table 2SI). To better visualize the effects of the various ligands on the peptide-bilayer interaction, Fig. 4 (panels D,F) shows the 2A_max_ variations of the ligand-bilayer systems upon the addition of A*β*(25–35).

Inspection of the figure indicates that the presence of ligands at the bilayer interface hinders any membrane penetration of the peptide. We propose that the accumulation of a layer of bound curcumin (or its analogues) at the lipid membrane interface reduces the accessibility and number of binding sites for A*β* on the membrane. Moreover, curcumin on the membrane surface can reduce A*β*–membrane interaction by altering the lipid head-group charge and reducing the favorable electrostatic interactions between A*β* and lipid head-groups^64^. The inspection of Fig. 3 clearly shows that all the ligands behave similarly, with the possible exception of CYC in DOPG membrane: in this case, the further 2A_max_ increase due to A*β*(25–35) suggests that the peptide preserves a detectable ability to interact with the bilayer even in the presence of the ligand. This evidence supports the hypothesis that DOPG membranes create a perfect environment to enhance A*β*(25–35) interaction with CYC.

### Molecular Modeling Studies

To relate the obtained CD and EPR results to the different structural features of the tested curcuminoids, a molecular modeling study was conducted. In particular, the structural features responsible for the interaction with A*β*(25–35) were investigated.

First, a comprehensive conformational analysis was performed on all possible tautomers of i) CUR, which is unable to affect the A*β*(25–35) conformational equilibrium either in a liposomal environment or in HFIP/water solution; ii) THC, which affects the A*β*(25–35) conformational equilibrium only in a liposomal environment; and iii) CYC, which perturbs the conformational behavior of A*β*(25–35), both, in DOPG and HFIP/water solution. The conformational space was sampled using a protocol including molecular dynamics (MD) simulations followed by molecular mechanic (MM) optimization, mimicking the polarity of a water medium (ε = 80*r). The identified minima were then subjected to full geometry optimization using the semi-empirical PM7 (MOPAC) method (ε = 1; for details see Experimental Section), thus obtaining a new set of conformers using a dielectric constant similar to the one for the highly hydrophobic membrane environment^65^. In this regard, it must be emphasized that only qualitative considerations can be deduced by comparing the results obtained from MM (ε = 80*r) and PM7 (ε = 1) calculations.

The MM and PM7 conformers obtained were ranked by their potential energy values (i.e., ΔE from the global energy minimum) and classified into different families based on the values of their torsional angles. Then, the distance between the centroids of the two aromatic rings was calculated (d1; Figures 3SI, 5SI–8SI; Tables 3SI–6SI). According to this latter parameter, the conformers were further classified as i) “folded” when d1 was ≤5 Å, ii) “semi-folded” when d1 was >5 Å and <9 Å, and iii) “extended” when d1 was ≥9 Å. Finally, the occurrence rate of each conformational family was calculated (Table 1 and Tables 3SI–6SI).

The results showed peculiar conformational features for each of CUR, CYC, and THC. The conformational behavior of CUR differed in the two tautomeric forms. Indeed, the conformers of the CUR-diketone form are distributed in two main conformational families: the “extended” and the “folded” (Tables 1 and 3SI; Figures 3SI–4SI), with the latter including the global minimum (GM) conformer. In particular, comparing PM7 with MM shows an increase in the number of “folded” conformers, i.e., presenting the two aromatic rings interacting with each other, and a concomitant decrease in the “extended” conformers can be observed. This result is due to the higher degree of accuracy of semi-empirical vs. MM methods in calculating electronic parameters, as well as to the fact that electrostatic interactions are expected to increase in non-polar (low dielectric) solvent.

**Figure 5:**
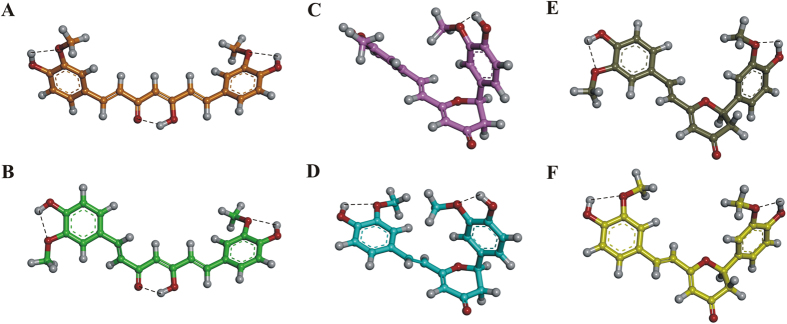
PM7 lowest energy conformers of i) keto-enol CUR conf I (**A**; orange) and conf II (**B**; green) and ii) CYC conf I (**C**; pink), conf II (**D**; cyan), conf III (**E**; gray), and conf IV (**F**; yellow). The conformers are displayed as ball & stick models and colored by atom type: O, red; H, white. Hydrogen bonds are highlighted with a black dashed line.

In contrast, in the keto-enol form of CUR, the conjugation of the double bonds extending from one aromatic ring to the other and the formation of a hydrogen bond between the ketone oxygen and the hydrogen of the enol function (Fig. 5A and B) strongly constrain the conformational freedom of the structure. Consequently, all CUR keto-enol conformers belong to the “extended” conformational family, resulting from both the MM and PM7 calculations (Tables 1 and 4SI; Figure 5SI). In detail, the PM7 low-energy conformers (within 5 kcal/mol from the GM) of the keto-enol form of CUR showed a specific preference for two sets of conformations, differing only for the value of the torsional angle τ1, herein named conf I (τ1 = ∼180°) and conf II (τ1 = ∼0°) (Fig. 5A and B; Table 4SI). These results are consistent with the ones obtained from the analysis of the experimentally determined structures of CUR (all in the keto-enol form) deposited in the Cambridge Structural Database (CSD) (Table 7SI). Indeed, CUR can assume either conf I (CSD codes: AXOGIE, AXOGOK, BINMEQ06, BINMEQ07, and BINMEQ08) or conf II (CSD codes: BINMEQ, BINMEQ01, BINMEQ02, BINMEQ03, BINMEQ04, and BINMEQ05), as shown by our conformational analysis.

**Figure 6:**
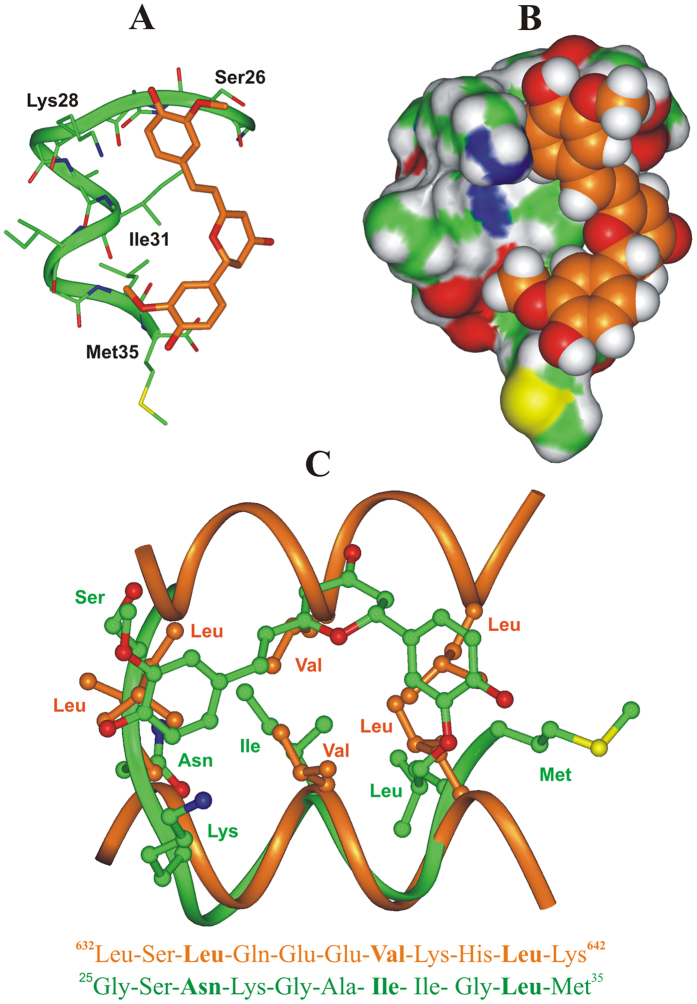
(**A**,**B**) Docked structure of CYC (orange) in complex with A*β*(25–35) peptide (green). (**A**) The complex is displayed as ribbon and sticks and peptide interacting residues are labeled; hydrogens are omitted for sake of clarity. (**B**) The Connolly surface of the peptide and the vdW volume of CYC are displayed. (**C**) X-ray structure of the coiled-coil dimerization motif of human ROCK I protein (carbon atoms: orange; PDB ID: 3O0Z) superimposed on the docked complex of CYC: A*β*(25–35) peptide (carbon atoms: green). Hydrogens are omitted for clarity. The corresponding 2-D sequence of human ROCK I protein and A*β*(25–35) are reported and the conserved residues of the heptad repeat motif are highlighted in bold. Molecules are colored by atom type (O: red; N: blue; S: yellow and H: white).

The presence of the dihydropyran-4-one ring in CYC determined a different conformational behavior from the observation for CUR tautomers. Indeed, all CYC conformers, both MM and PM7, fall into the “semi-folded” conformational family (Table 1 and Figure 6SI). Overall, CYC showed four possible subfamilies of conformers, differing for the values of the torsional angles τ1, τ2, and τ3 and characterized by d1 ranging from 7.00 to 9.66 Å (conf I–IV in Fig. 5C–F and Table 5SI).

THC shows yet another conformational preference compared either to CUR or CYC. According to the results of our analysis, THC conformers fall into all three conformational families regardless of the tautomeric form or the computational method used (Table 1; Figure 9SI; Table 6SI). However, either in the diketone or in the keto-enol form, the “folded” conformation is strongly stabilized by the formation of a hydrogen bond between the hydroxyl groups of the two aromatic rings (Figure 9SI). This intra-molecular H-bond may compete with the formation of H-bond interactions with solvent molecules, which are not treated explicitly in this simulation. Thus, it is likely that the actual conformational behavior of THC strongly depends on the chemical environment.

Furthermore, to investigate the experimentally observed interaction between A*β*(25–35) and CYC in HFIP/water 80/20 solution, we performed a dynamic molecular docking simulation on the CYC-A*β*(25–35) complex, mimicking the polarity of the media with a dielectric constant of 30*r. To fully explore all possible binding sites/modes during the dynamic docking procedure, the binding site was defined as the whole peptide, and all rotatable bonds of CYC and A*β*(25–35) were left fully free to move. A force constant of 10 kcal/mol/Å^2^ was only applied on the backbone hydrogen bonds of (Asn27-Leu34) to avoid unrealistic results during the annealing simulation (for details, see the Experimental Section). First, a Monte Carlo/minimization approach for the random generation of a maximum of 20 acceptable complexes was used. To ensure a wide variance of the input structures to be minimized afterward, an energy tolerance value of 10^6^ kcal/mol from the previous structure was used. After the energy minimization step, the energy test, with an energy range of 50 kcal/mol, and a structure similarity check (rms tolerance = 0.3 kcal/A) was applied to select the 20 acceptable structures. To test the thermodynamic stability of the resulting docked complexes, these latter were then subjected to simulated annealing (SA) calculations. In SA the temperature is altered in time increments from an initial (500 K) to final (300 K) temperature. The temperature of 500 K was applied with the aim of surmounting torsional barriers, thus allowing a full rearrangement of the ligand and the peptide (see Experimental section for details).

The resulting complexes were analyzed and ranked by their conformational and interaction energy values (Table 8SI). On twenty Monte Carlo generated structures, only twelve (Figure 10SI) still present CYC bound to A*β*(25–35) peptide after SA calculations. Interestingly, just one of these twelve SA complexes preserves the same binding mode resulting from Monte Carlo docking procedure, showing thermodynamic stability (Complex 1, Figure 10SI). Importantly, this complex also resulted to be the lowest energy complex and the one characterized by the most favorable interaction energy (Table 8SI). Such results strongly suggest that Complex 1 is representative for a stable and rather specific binding mode of CYC to A*β* (25–35) peptide (Fig. 6A and B).

Interesting data provided by our docking results showed that the calculated binding mode of CYC to A*β*(25–35) resembles the canonical packing of the α-helical coiled-coil dimerization motif, where the side-chains of the “a” and “d” residues of two facing heptad repeat motifs interact with each other (Fig. 6C)^66^. In fact, in the consensus sequence of the heptad repeat motif, the “a” and “d” positions are occupied by hydrophobic residues (one of which can be replaced by an asparagine), favoring the formation of coiled-coil structures, where the i, i + 4, and i + 7 residues of a helix interact with the equivalent residues of another helix. As evidenced by the superimposition reported in Fig. 6C, CYC can mimic the interactions performed by such residues. In particular, the two substituted phenyl rings (d1 = 8.85 Å) can reproduce the orientation of the hydrophobic side chains of the i and i + 7 residues (distance from the side chain centroids = 8.20 Å), and at the same time, the dihydropyran-4-one ring and the ethylene bridge overlap with the central i + 4 residue (Fig. 6C).

Taken together our computational results support the hypothesis that CYC targets heptad repeat motifs like the one found in A*β*(25–35) (Fig. 6C), whose structure is preserved in full length A*β*(1–42) and which is part of the extended heptad repeat motif identified as responsible for the dimerization of APP in micellar environment (Figure 11SI)^67^.

## Discussion

A great deal of evidence shows that curcumin and several synthetic curcumin derivatives bind beta amyloid peptides, affecting the process of fibril formation^31^,^32^,^33^,^34^,^35^,^36^. In light of the well-documented pharmacological effects, including the neuroprotective effect exerted by the less abundant curcuminoid molecules in the *Curcuma Longa* extract, we studied these molecules for their ability to affect the conformational arrangement of A*β*(25–35), providing a molecular basis for the observed biological effect.

In particular, our analyses were intended to understand whether the neuroprotective effect of the phytocomplex derived from *Curcuma Longa* can be credited to the ability of the curcuminoids to lessen lipid membrane-A*β* interactions and, by consequence, the A*β*-induced membrane-disruptive perturbations^49^,^50^,^51^. Accordingly, we investigated how curcuminoids, taken as single purified molecules, affect A*β*(25–35)−membrane interactions through CD and EPR experiments, using DOPC, DOPG and DOPC/DOPG liposomal solutions to mimic lipid bilayer membrane systems. The CD spectra of monomeric A*β*(25–35) in the different superficially charged liposome environments were recorded after the addition of the compounds CUR, DMC, BDMC, CYC, THC, HHC, OHC and MIX. These experiments were conducted to evaluate the effect of curcuminoids in solution on A*β*(25–35)-membrane interactions. In the presence of these curcuminoids, used pure or mixed, no significant modification of the A*β*(25–35) conformational equilibrium was observed in DOPC and DOPC/DOPG liposomal solution. In contrast, when CYC and THC were inserted into anionic DOPG lipid solution, a significant modification of the A*β*(25–35) CD curves was detected, corresponding to a perceptible increase in the soluble form of the *α*-helix vs. *β*-strand and random coil conformations. These data were obtained by EPR experiments that revealed, for both A*β*(25–35) and curcuminoids, a shallow insertion in the DOPC, DOPC/DOPG, and DOPG lipid layers, just below the outer interface (5-PCLS 2A_max_ shift). Only in the case of CYC in DOPG liposomal solution was a slight but significant perturbation of 14-PCSL 2A_max_ observed, demonstrating that the molecule reaches the inner part of the bilayer. The inclusion of the curcuminoid ligands on the bilayers prevents further interaction of the membrane with the A*β* peptide, causing only minimal variation in the ligand-bilayer systems, with the sole exception of CYC in DOPG. These results are consistent with other literature data, based on TEM (transmission electron microscopy) images and CD spectra^64^, which also evidenced that the presence of CUR does not inhibit A*β*40 aggregation or significantly alter the morphologies of A*β* monomers, prefibrillar or fibrillar aggregates, while it reduces the extent of cell membrane permeabilization induced by A*β* aggregates.

In the case of DOPG membrane containing CYC or THC, the presence of the guest molecules induces a significant alteration of the A*β*(25–35) conformational equilibrium, as observed in the CD spectra, and a marked perturbation of the membrane outer layer, as observed in the EPR experiments. This result may indicate a direct interaction of CYC and THC with A*β*(25–35), but it still cannot be excluded that the observed effects are mediated by the lipid membrane. To investigate this issue, CD spectra were recorded in an HFIP/water (80/20 v:v) mixture in the absence and in the presence of CYC and THC. Under these conditions, CYC was still able to modify the conformational equilibrium of A*β*(25–35) (Fig. 3C,D), whereas THC induced only minimal modification of the A*β*(25–35) secondary structure. Thus, THC does not directly interact with monomeric A*β*(25–35) in solution; nonetheless, when integrated into the DOPG system, it induces a modification in the A*β*(25–35) conformational equilibrium mediated by the lipid membrane. By contrast, CYC can modify the conformation of A*β*(25–35) either in HFIP/water solution or in anionic DOPG lipid solution, indicating that its constrained structure - naturally derived from an intramolecular cyclization - is suitable for direct interaction with A*β*(25–35) in both environments. Computational studies provided a molecular model for this interaction and a possible explanation for the peculiar ability of CYC, unlike CUR and THC, to interact directly with A*β*(25–35), either in solution or in the liposomal environment. Indeed, according to the results of our molecular modeling studies, this result is due to the constrained “semi-folded” conformation of CYC, which allows it to mimic the orientation of the hydrophobic side chains of short peptide motifs, similarly to our previous observations for other small ligands^68^,^69^. Thus, by mimicking the orientation of the i, i + 4, and i + 7 residues of an α-helix, CYC could interact with A*β*(25–35), reproducing the pattern observed in α-helical coiled-coil structures, the latter being characterized by the presence of one of the principal dimerization motifs in proteins^70^. Coiled-coil peptide structures are known to anchor to cellular membranes^71^ and are formed by heptad repeats containing hydrophobic residues at the “a” and “d” positions. Importantly, the formation of helical coiled-coil structures, through the reciprocal interaction of several heptad repeat motifs, was also observed for APP in micelles, leading to its dimerization^67^. In line with these results, our previous NMR studies evidenced a strong similarity between the structure of A*β*(1–42) peptide in an apolar microenvironment and the structure of the fusion domain of influenza hemagglutinin in detergent micelles (also characterized by the ability to form coiled-coil structures)^72^. A heptad repeat motif is still contained in the A*β*(25–35) fragment, and, similarly to previous observations, could lead to the formation of α-helical coiled-coil structures on the membrane lipid surface that are amenable to interaction with CYC. On the other hand, monomers and soluble aggregates of A*β*(25–35) in the form of α-helix are also likely to occur in HFIP/water solution, since, in this environment, the helical structure of A*β*(25–35) is strongly stabilized^73^. Accordingly, CYC also perturbs the conformational equilibrium of A*β*(25–35) in this environment.

The “semi-folded” conformation of CYC is poorly present in CUR (not present at all in its predominant keto-enol form) but still significantly present in the case of THC. However, due to the higher conformational flexibility of THC with respect to CYC, it represents just a fraction of the whole conformational space, with its relative weight depending on the polarity and the H-bond donor/acceptor properties of the solvent. Accordingly, CUR and other analogues characterized by a conjugated and mostly planar structure (DMC, BDMC) behave only as membrane stabilizers, forming ideal mixtures with DOPG lipids, reducing lipid flexibility, and preventing A*β*(25–35)-peptide insertion. On the contrary, CYC, which is structurally constrained in the “semi-folded” conformation, is able to bind/stabilize the monomeric helical form of A*β*(25–35). In this way, CYC may prevent not only the formation of toxic *β*-sheet aggregate, but also the formation of membrane disrupting aggregates formed by α-helical coiled-coil structures^74^,^75^. Finally, the more flexible THC shows a different behavior in DOPG bilayer or in the HFIP/water mixture; indeed, THC shifts the conformational equilibrium of A*β*(25–35) to the helical structure in DOPG, but this ability was not preserved in the HFIP/water solution.

## Conclusions

The results of this work show that the well-documented biological activity of turmeric extract is based on a molecular mechanism in which CUR and its analogues reduce the A*β*–membrane interactions due to their marked tendency to interact with the lipid bilayer. However, this effect is differently mediated among the examined curcuminoids since i) only CYC and THC can modify the conformational behavior of A*β*(25–35) in the liposome environment, stabilizing the α-helix conformation and ii) only CYC can still modify the conformational behavior of A*β*(25–35) in the HFIP/water mixture. While the enol form of CUR (“extended” conformation) was found to be responsible for the inhibition of A*β* fibril formation, our molecular modeling studies suggest that, to interact with the helical form of A*β* and prevent the formation of membrane-disrupting aggregates (possibly consisting of α-helical coiled-coil structures), it is necessary for CUR and its derivatives to assume the partially folded conformation in which CYC is naturally constrained.

This combined approach leads to a better understanding of the intriguing *in vitro* and *in vivo* activity of curcuminoids as anti-Alzheimer agents, paving a new path for the rational design of optimized druggable analogues.

## Experimental Section

All reagents and solvents were purchased from Sigma-Aldrich (St. Louis, MO, USA) and used as received. Curcumin was supplied from Indena S.p.A. (Milano, Italy) as mixture of curcuminoids extracted from *Curcuma Longa*. The phospholipids 1,2-dioleoyl-*sn-*glycero-3-phosphocholine (DOPC) and 1,2-dioleoyl-*sn-*glycero-3-phospho-(1′-rac-glycerol) (sodium salt) (DOPG) were purchased from Avanti Polar Lipids (Birmingham, AL, USA). All microwave reactions were conducted in a CEM Explorer^®^ apparatus under monomode irradiation. Hydrogenation of curcuminoids was performed in a Parr^®^ apparatus. All reactions were conducted under dry nitrogen atmosphere. Flash column chromatography was performed on silica gel 60, 0.040 −0.063 mm (230–400 mesh) using methanol/dichloromethane solvent mixtures. Silica gel (grade 60 PF254) was used for preparative TLC. An Agilent 1260 high-performance liquid chromatography system with a variable wavelength detector was used for analysis. NMR spectra were recorded on a 300 MHz NMR spectrometer using CDCl_3_ and MeOD as the solvent and TMS as the internal standard, unless otherwise specified. Conditions are specified for each spectrum (temperature 25 °C unless otherwise specified). Splitting patterns are designated as *s*, singlet; *d*, doublet; *t,* triplet; *q*, quartet; *m*, multiplet. Chemical shifts (*δ*) are given in ppm relative to the resonance of their respective residual solvent peak, MeOH (4.87 ppm, ^1^H; 49.00 ppm, the middle peak, ^13^C). High-resolution mass spectra (HRMS) were recorded on a high-resolution mass spectrometer equipped with electrospray (ESI) and nanospray sources and a quadrupole-time of flight hybrid analyzer coupled with a capillary UPLC system (Q-TOF Premier/nanoAquity, Waters) in positive mode, and protonated molecular ions [M + H]^+^ were used to confirm the empirical formula, unless otherwise stated.

### Separation of curcuminoids

Liquid chromatography was performed on a Waters system (Milford, Massachusetts, USA) consisting of a Waters 486 Tunable Absorbance Detector and a Varian 9012 pump. Samples were prepared by dissolving curcuminoids (CUR, DMC, BDMC, CYC) and MIX in acetonitrile at 0.2 mL/min. The injection volume was 10 μL. Curcuminoids were separated on a Teknokroma C18 column (25 × 0.46 cm, 5 μm) under gradient elution at a flow rate of 0.9 mL/min. The mobile phases consisted of 2% (v/v) acetic acid in water (solvent A) and 2% (v/v) acetic acid in acetonitrile (solvent B). The following gradient was used: 0–20 min 30–70% B, 20–28 min 70–90% B, and return at initial conditions in 2 min. Detection UV at λ = 254 nm.

### Curcumin (1)

Odorless orange powder, MP: 178–180 °C; ^1^H NMR (300 MHz, MeOD) δ 7.58 (d, *J* = 15.9 Hz, 2 H), 7.22 (s, 2 H), 7.11 (d, *J* = 7.4 Hz, 2 H), 6.82 (d, *J* = 7.7 Hz, 2 H), 6.63 (d, *J* = 15.7 Hz, 2 H), 5.97 (s, 1 H), 3.91 (s, 6 H) ppm. ^13^C NMR (75 MHz, MeOD) δ 185.63, 151.14, 150.07, 142.71, 129.10, 124.66, 122.76, 117.01, 112.26, 112.04, 56.50, 56.02 ppm. HRMS (ESI-Q-TOF) m/z [M + H]^+^ Calcd. for C_21_H_21_O_6_ 369.1333; Found 369.1335.

### Demethoxycurcumin (2)

Odorless orange powder, MP: 157–161 °C; ^1^H NMR (300 MHz, MeOD) δ 7.52 (dd, *J* = 26.6, 11.4 Hz, 4 H), 7.20 (s, 1 H), 7.09 (d, *J* = 7.7 Hz, 1 H), 6.81 (d, *J* = 7.4 Hz, 3 H), 6.73–6.49 (m, 2 H), 3.90 (s, 3 H) ppm. ^13^C NMR (75 MHz, MeOD) δ 185.71, 185.51, 162.05, 151.38, 150.11, 142.68, 142.45, 131.67, 128.94, 128.36, 124.61, 122.62, 122.36, 117.39, 117.05, 112.21, 111.99, 101.97, 56.47 ppm. HRMS (ESI-Q-TOF) m/z [M + H]^+^ Calcd. for C_20_H_19_O_5_ 339.1227; Found 339.1230.

### Bisdemethoxycurcumin (3)

Odorless orange powder, MP: 187–191 °C; ^1^H NMR (300 MHz, MeOD) δ 7.58 (d, *J* = 16.1 Hz, 3 H), 7.49 (d, *J* = 8.4 Hz, 4 H), 6.82 (d, *J* = 8.3 Hz, 5 H), 6.60 (d, *J* = 15.9 Hz, 2 H), 2.17 (d, *J* = 7.1 Hz, 3 H) ppm. ^13^C NMR (75 MHz, MeOD) δ 185.69, 161.94, 142.43, 131.67, 128.46, 122.44, 117.36. HRMS (ESI-Q-TOF) m/z [M + H]^+^ Calcd. for C_19_H_17_O_4_ 309.1121; Found 309.1117.

## Procedures for the synthesis of compounds 4, 5, 6, 7

### Cyclocurcumin (4)

In a 7 mL MW vessel, curcumin **1**, (20 mg, 0.054 mmol) and TFA (2.14 mmol, 164 μL) were added, and the reaction mixture was stirred in CEM Explorer^®^. MW Method: T = 100 °C, Power: 300 W, Hold Time: 4 min, P = 250 PSI, Power Max activated. After cooling, TFA was removed under reduced pressure, and the residue was purified by flash column chromatography (CH_2_Cl_2_/MeOH 97:3) to afford cyclocurcumin **4** as a yellowish oil (2 mg, 10%). ^1^H NMR (300 MHz, MeOD) δ 7.35 (d, *J* = 15.8 Hz, 1 H), 7.18 (s, 1 H), 7.12 (d, *J* = 1.7 Hz, 1 H), 7.05 (d, *J* = 8.3 Hz, 1 H), 6.99 (d, *J* = 8.2 Hz, 1 H), 6.86 (d, *J* = 8.1 Hz, 1 H), 6.77 (d, *J* = 8.2 Hz, 1 H), 6.66 (d, *J* = 15.8 Hz, 1 H), 5.62 (s, 1 H), 5.45 (dd, *J* = 13.7, 3.2 Hz, 1 H), 3.89 (d, *J* = 7.3 Hz, 6 H), 3.03 (dd, *J* = 16.9, 13.8 Hz, 1 H), 2.59 (dd, *J* = 16.9, 3.0 Hz, 1 H). ^13^C NMR (75 MHz, MeOD) δ 197.20, 150.27, 143.79, 142.37, 131.77, 124.74, 124.41, 122.41, 121.35, 119.44, 117.23, 116.70, 112.06, 111.87, 94.18, 83.43, 82.57, 56.47, 55.71 ppm. HRMS (ESI-Q-TOF) m/z [M + H]^+^ Calcd. for C_21_H_21_O_6_ 369.1333; Found 369.1331.

### Tetrahydrocurcumin (5)

A solution of curcumin **1** (50 mg, 0.14 mmol) in dry MeOH (5.9 mL) was introduced into an oven-dried bottle connected to a Parr apparatus. Pt/C 10% wt (2.14 mg, 0.014 mmol) was added, and the bottle was filled with H_2_ (1 atm) and shaken at room temperature for 1 h. The bottle was degassed, and the catalyst was filtered (*ATTENTION: the Pt residue might be pyrophoric*) and washed several times with MeOH. The MeOH fractions were collected, and the solvent was removed under reduced pressure to give compound **5** as a pale white oil (40 mg, 77% yield). ^1^H NMR (300 MHz, MeOD) δ 6.80–6.55 (m, 6 H), 3.80 (s, 6 H), 2.78 (dd, *J* = 15.5, 7.6 Hz, 6 H), 2.53 (t, *J* = 7.5 Hz, 3 H) ppm. ^13^C NMR (75 MHz, MeOD) δ 195.58, 149.46, 146.42, 134.21, 122.18, 116.55, 113.47, 56.38, 46.25, 41.27, 32.27, 29.93 ppm. HRMS (ESI-Q-TOF) m/z [M + H]^+^ Calcd. for C_21_H_25_O_6_ 373.1646; Found 373.1648.

### Hexahydrocurcumin (6) and octahydrocurcumin (7)

A solution of curcumin **1** (100 mg, 0.27 mmol) in dry MeOH (11.8 mL) was introduced into an oven-dried bottle connected to a Parr apparatus. Pt/C 10% wt (5.27 mg, 0.027 mmol) was added, and the bottle was filled with H_2_ (6.8 atm) and shaken at room temperature for 13 h. The bottle was degassed, and the catalyst was filtered (*ATTENTION: the Pt residue might be pyrophoric*) and washed several times with MeOH. The MeOH fractions were collected, and the solvent was removed under reduced pressure to give compounds **6** and **7** as a mixture (*ratio* 40:60). The crude mixture was purified by flash column chromatography (CH_2_Cl_2_/MeOH 97:3) to afford **6** (37 mg, 37% yield) and **7** (56 mg, 55% yield) as pale white oils. *Hexahydrocurcumin (**6***). ^1^H NMR (300 MHz, MeOD) δ 6.75 (s, 7 H), 6.69 (dd, *J* = 8.0, 3.0 Hz, 11 H), 6.60 (d, *J* = 8.1 Hz, 11 H), 4.00 (dd, *J* = 11.2, 5.8 Hz, 5 H), 3.81 (d, *J* = 4.0 Hz, 32 H), 2.83–2.67 (m, 21 H), 2.57 (ddd, *J* = 15.7, 12.6, 6.3 Hz, 22 H), 1.68 (dd, *J* = 13.8, 7.6 Hz, 14 H) ppm. ^13^C NMR (75 MHz, MeOD) δ 212.95, 149.48, 146.33, 146.13, 135.41, 134.56, 122.24, 122.14, 116.53, 113.56, 113.50, 68.87, 68.34, 67.80, 56.89, 56.55, 56.39, 56.22, 51.29, 46.32, 40.37, 32.28, 30.13 ppm. HRMS (ESI-Q-TOF) m/z [M + H]^+^ Calcd. for C_21_H_27_O_6_ 375.1802; Found 375.1805. *Octahydrocurcumin (**7***). ^1^H NMR (300 MHz, MeOD) δ 6.83–6.46 (m, 3 H), 3.78 (dd, J = 20.0, 3.4 Hz, 4 H), 2.76–2.41 (m, 2 H), 1.82–1.44 (m, 3 H) ppm. ^13^C NMR (75 MHz, MeOD) δ 149.44, 146.06, 135.70, 122.22, 116.51, 113.54, 71.12, 68.83, 56.50, 44.81, 40.73, 32.21 ppm. HRMS (ESI-Q-TOF) m/z [M + H]^+^ Calcd. for C_21_H_29_O_6_ 377.4508; Found 377.4506.

### Peptide synthesis

The A*β*(25–35) peptide, (G-S-N-K-G-A-I-I-G-L-M), was manually synthesized by conventional solid-phase peptide synthesis (SPPS) using the Fmoc/tBu strategy^76^. The Nα-Fmoc-protected amino acids were coupled using 1-hydroxybenzotriazole and *O*-(benzotriazol-1-yl)-1,1,3,3-tetramethyluroniumhexa-fuorophosphate (fourfold excess) as coupling reagents, in the presence of a six-fold excess of *N,N*-diisopropylethylamine. Peptide-resin (Wang resin) cleavage and side-chain deprotection reactions were performed in 90% trifluoroacetic acid (TFA), 5% water and 5% triisopropylsilane (TIS). The resin was filtered and the solution added to cold diethyl ether to precipitate the peptide.

Subsequently, the peptide was purified by reversed phase chromatography (HPLC) using a Phenomenex column C18 (30 cm, 4 cm, 300 Å, 15 mm spherical particle size column). The peptide was characterized on a Finningan LCQ Deca ion trap instrument equipped with an electrospray source (LCQ Deca Finnigan, San José. CA, USA). The samples were directly infused in the ESI source using a syringe pump at a flow rate of 5.0 mL/min. The data were analyzed using the Xcalibur software. The sample purity was >98%.

### Sample preparation for CD analysis

All CD experiments were recorded in small unilamellar liposome vesicles (SUVs) consisting of pure DOPC lipid, pure DOPG lipid and DOPC/DOPG mixtures at 90/10 M/M (molar ratio). Samples of DOPC, DOPG and DOPC/DOPG (90/10) small unilamellar liposome vesicles (SUVs) were prepared as follows: 200 μM of each phospholipid were dissolved in a CH_2_Cl_2_/MeOH solution, then kept in a round-bottom test tube, and a thin lipid film was produced by evaporating the solvent with dry nitrogen gas. The final traces of solvent were removed by subjecting the sample to vacuum desiccation for one night. The samples were then hydrated with an appropriate volume of 10 mM phosphate buffer at pH = 7.4 and repeatedly vortexed, obtaining a suspension. The MLV suspension thus obtained was sonicated for 5–10 minutes to obtain SUVs.

To remove aggregate states, which can be present in A*β* samples, the dry peptide was pretreated with TFA for 3 hours, followed by dilution with MilliQ water and lyophilization. The TFA pretreatment gives the A*β* peptide the properties of monomeric, random coil structures and eliminates preaggregated material^77^. This procedure was adopted for all CD samples, immediately before dissolution in the appropriate solvent. This procedure was also adopted before preparing A*β*(25–35) amyloid fibrils^78^.

To record CD experiments, A*β*(25–35) that had been previously treated according to the procedure reported above was added to SUVs (DOPC, DOPG and DOPC/DOPG (90/10)) to have a final peptide concentration of 0.1 mM (CD experiments) and a peptide-lipid molar ratio of 0.5:1 for each experiment. Similarly, ligands (CUR, DMC, BDMC, MIX, CYC, THC, HHC and OHC) were added to SUVs (DOPC, DOPG and DOPC/DOPG (90/10)) for a final ligand concentration of 0.5 mM (CD experiments).

### CD measurements

Circular dichroism measurements were performed on a JASCO J-810 spectropolarimeter (Jasco, Cremella, Italy) equipped with a thermostated cell holder, using a quartz cell with a 1.0-mm path length. Spectra were collected over the wavelength range from 260–190 nm with a bandwidth of 2.0 nm and a time constant of 8 s.

All CD spectra were recorded in three different liposome vesicles (DOPC, DOPG and DOPC/DOPG (90/10)) to which A*β*(25–35) was added at a final concentration of 0.1 mM and all ligands at a final concentration of 0.5 mM. The CD spectrum of A*β*(25–35) in the absence of ligands was recorded for comparison. Each spectrum was corrected for the contribution of the liposomes containing pure ligands. To estimate the secondary structure, the CD spectra were analyzed using the CONTINN, SELCON and K2D algorithms of the DICHROWEB on-line server^57^.

### EPR spectroscopy

For EPR experiments, multi-lamellar vesicles (MLVs) of DOPC, DOPG and DOPC/DOPG (90/10 molar ratio) were prepared by mixing appropriate amounts of lipids, dissolved in a CH_2_Cl_2_/MeOH mixture (2:1 v/v, 10 mg/mL concentration), in a round-bottom test tube. Spin-labeled phosphatidylcholines (1-palmitoyl-2-stearoyl-(n-doxyl)-*sn*-glycero-3-phosphocholine, n-PCSL with n = 5, 14) were added to the lipid mixture (1% by weight of the total lipid) by mixing appropriate amounts of a spin-label solution in ethanol (1 mg/mL) with the lipid organic mixture. A thin lipid film was produced by evaporating the solvents with dry nitrogen gas. The total weight of the lipids for each sample was 0.2 mg. The final traces of solvents were removed by subjecting the sample to vacuum desiccation for at least 3 h. The samples were then hydrated with 20 μL of 10 mM phosphate buffer at pH = 7.4, gently warmed and repeatedly vortexed to obtain a MLV suspension.

Samples containing curcumin or one of its analogues were prepared by the same procedure, adding appropriate amounts of the flavonoid dissolved in DMSO (10 mg/mL) to the buffer during the re-hydration step to obtain a 0.5 molar percentage with respect to the total lipids. The addition of DMSO was preventively checked to result in no perturbation of the vesicular systems.

Samples containing A*β*(25–35) were prepared in a similar manner, except that the lipid film was hydrated directly with the peptide solution in phosphate buffer, at 1:0.5 lipid/peptide weight ratio (which corresponds to approximately 20:1 mol/mol).

EPR spectra were recorded with a 9 GHz Bruker Elexys E-500 spectrometer (Bruker, Rheinstetten, Germany). Samples were placed in 25 μL glass capillaries and flame sealed. The capillaries were placed in a standard 4 mm quartz sample tube containing light silicone oil for thermal stability. All measurements were performed at 25 °C. The spectra were recorded using the following instrumental settings: sweep width, 100 G; resolution, 1024 points; time constant, 20.48 ms; modulation frequency, 100 kHz; modulation amplitude, 1.0 G; incident power, 6.37 mW. Several scans, typically 16, were accumulated to improve the signal-to-noise ratio.

### Molecular modeling

Molecular modeling calculations were performed on an SGI Origin 200 8XR12000 and an E4 Server Twin 2 x Dual Xeon 5520, equipped with two nodes. Each node was 2 x Intel Xeon QuadCore E5520, 2.26 GHz, 36 GB RAM. The molecular modeling graphics were implemented on SGI Octane 2 workstations.

The apparent pKa values of CUR and its derivatives in their tautomeric forms were estimated using the ACD/Percepta software^79^. Accordingly, the percentages of neutral/ionized forms were computed at pH 7.2 (cytoplasm) using the Henderson-Hasselbach equation.

#### Conformational analysis

All compounds were built considering the prevalent ionic forms of each tautomer using the Insight 2005 Builder module (Accelrys Software, Inc., San Diego).

Atomic potentials and partial charges were assigned using the CVFF force field^80^. The conformational space of the compounds was sampled through 200 cycles of simulated annealing (ε = 80*r). To avoid unrealistic results, the torsional angle of the double bonds was constrained within 180° using a force constant of 100 (kcal/mol)/Å. In simulated annealing, the temperature is altered in time increments from an initial temperature to a final temperature by adjusting the kinetic energy of the structure (by rescaling the velocities of the atoms). The following protocol was applied: the system was heated to 1000 K over 2000 fs (time step of 3.0 fs); the temperature of 1000 K was applied to the system for 2000 fs (time step of 3.0 fs) to surmount torsional barriers; then, the temperature was linearly reduced to 300 K in 1000 fs (time step of 1.0 fs). The resulting conformations were then subjected to Molecular Mechanics (MM) energy minimization within the Insight 2005 Discover 3 module (CVFF force field; ε = 80*r) until the maximum RMS derivative was less than 0.001 kcal/Å, using the conjugate gradient^81^ as the minimization algorithm. The resulting MM conformers were ranked by (i) their potential energy values (i.e., ΔE from the global energy minimum), (ii) intra-molecular hydrogen bonds, (iii) torsional angles, and (iv) the interatomic distance between the centroids of the two aromatic rings. The centroids were determined considering the heavy atoms of the aromatic rings (Pseudo_Atom Define command, Biopolymer Module, Insight 2005). Then, the occurrence rates were calculated.

All MM conformers were subjected to a full geometric optimization by semi-empirical calculations, using the quantum mechanical method PM7^82^ in the MOPAC2012 package^82^ and EF^83^ (eigenvector following routine) as the geometric optimization algorithm. The GNORM value was set to 0.01. To achieve full geometric optimization, the criterion for terminating all optimizations was increased by a factor of 100, using the keyword PRECISE. The resulting PM7 conformers were ranked as MM conformers.

Finally, the X-ray structures of CUR, alone (CSD codes: AXOGIE, AXOGOK, BINMEQ, BINMEQ01, BINMEQ02, BINMEQ03, BINMEQ04, BINMEQ05, BINMEQ06, BINMEQ07, BINMEQ08, BUWKUZ, GANJAG) or in complex with any metal (CSD code: RAWDOK), were selected and downloaded from the Cambridge Structural Database (CSD) using the CSDS (Cambridge Structural Database System) software Conquest 1.16. These structures were analyzed using Insight 2005 (Accelrys, San Diego, CA).

#### Docking procedure

To build the starting complex CYC:A*β*(25–35) for dynamic docking studies, the experimentally determined structure of A*β*(25–35) (PDB ID: 1QWP) was downloaded from the Protein Data Bank (PDB; http://www.rcsb.org/pdb/). Hydrogens were added to the PDB structure assuming a pH of 7.2. This structure was analyzed and compared with the experimentally determined structures of HDM2 protein in complex with p53 (PDB ID: 1YCR), using the Biopolymer and Homology module of Insight 2005 (Accelrys, San Diego). Accordingly, a structural superimposition was performed between the α-helix of HDM2 (A50-A65) and Aβ(25–35). The centroids of interacting residues of considered LxxLLxxL-like (i, i + 3, i + 4, i + 7) motif (B17-B29; PDB ID: 1YCR) were built, and their relative distances were calculated. The resulting values were crossed with the values of the CYC pharmacophore distances. The lowest-energy PM7 conformer of CYC was superimposed on residues i and i + 4 (i.e., Phe19 and Trp23) of the LxxLLxxL-like motif by fitting the centroids of the aromatic rings (i.e., X and Y) on the centroids of the side chains of the corresponding residues. Then, the resulting structure of the complex CYC: A*β*(25–35) was used for successive dynamic docking studies. In particular, a docking methodology was used that treats all the systems (i.e., ligand and protein) as flexible. Flexible docking was achieved using the Affinity module in the Insight 2005 suite, setting the SA_Docking procedure^84^ and using the Cell Multipole method for nonbonding interactions^85^. To mimic the experimental conditions (a mixture of HFIP and water; 80/20 v:v), throughout the calculations, a dielectric constant of 30*r was used. This value was obtained as the weighted average of the dielectric constants of water (ε = 80) and HFIP (ε = 16.7).

The docking protocol included a Monte Carlo-based conformational search of the ligand (CYC) within the A*β*(25–35) peptide. The binding domain area was defined as a subset including all residues of the A*β*(25–35) peptide. All atoms included in the binding domain area were left free to move throughout the course of the docking calculations, whereas to avoid unrealistic results, a tethering restraint was applied to the SCRs of the protein. In particular, for the α-helix Asn27-Leu34, the distance between backbone hydrogen bond donors and acceptors was restrained within 2.5 Å using a force constant of 10 kcal/mol/Å^2^.

A Monte Carlo/minimization approach for the random generation of a maximum of 20 acceptable complexes was used. During the first step, starting from the previously obtained roughly docked structures, the ligand was moved by a random combination of translation, rotation, and torsional changes to sample both the conformational space of the ligand and its orientation with respect to the protein (MxRChange = 3 Å; MxAngChange = 180°). During this step, the van der Waals (vdW) and Columbic terms were scaled to a factor of 0.1 to avoid very severe divergences in the vdW and Columbic energies. If the energy of a complex structure resulting from random moves of the ligand was higher by the energy tolerance parameter than the energy of the last accepted structure, it was not accepted for minimization. To ensure a wide variance in the input structures to be successively minimized, an energy tolerance value of 10^6^ kcal/mol from the previous structure was used. After the energy minimization step (conjugate gradient; 10000 iterations; ε = 30*r), the energy test, using an energy range of 50 kcal/mol, and a structure similarity check (rms tolerance = 0.3 kcal/Å) were applied to select the 20 acceptable structures. Each subsequent structure was generated from the last accepted structure. Following this procedure, the resulting docked structures were ranked by their conformational energy and analyzed. Finally, to test the thermodynamic stability of the resulting docked complexes; these latter were subjected also to a molecular dynamics simulated annealing protocol (ε = 30*r). A tethering restraint was applied to the SCRs of the complex. The same set of structural restraints used for previous docking calculations was applied. The protocol included 5 ps of a dynamic run divided into 50 stages (100 fs each), during which the temperature of the system was linearly decreased from 500 to 300 K (Verlet velocity integrator; time step = 1.0 fs). In simulated annealing, the temperature is altered in time increments from an initial temperature to a final temperature. The temperature is changed by adjusting the kinetic energy of the structure (by rescaling the velocities of the atoms). Molecular dynamics calculations were performed using a constant temperature and constant volume (NVT) statistical ensemble, with direct velocity scaling as the temperature control method (temp window = 10 K). In the first stage, the initial velocities were randomly generated from the Boltzmann distribution, according to the desired temperature, while during the subsequent stages, the initial velocities were generated from the dynamics restart data. A temperature of 500 K was applied to surmount torsional barriers, thus allowing an unconstrained rearrangement of the “ligand” and the “protein” active site (initial vdW and Columbic scale factors = 0.1). The temperature was then linearly reduced to 300 K in 5 ps, and, concurrently, the vdW and Columbic scale factors were similarly increased from their initial values (0.1) to their final values (1.0). A final round of 10^5^ minimization steps (ε = 30*r) followed the last dynamics steps, and the minimized structures were saved in a trajectory file.

The resulting complexes were ranked by their conformational energy and analyzed by considering the non-bond interaction energies between the ligand and the peptide (vdW and electrostatic energy contribution; Group Based method; CUT_OFF = 100; ε = 2*r; Discover_3 Module of Insight2005). The complex characterized by the lowest conformational and interaction energy was chosen as the most representative. To allow the relaxation of the whole system, this complex was further minimized with no tethering restraint (CVFF force field; Cell Multipole method for non-bond interaction) using the Conjugate Gradient algorithm (maximum rms derivative less than 0.01 kcal/Å). Finally, the selected complex was compared with the experimentally determined structures of i) HDM2 protein in complex with p53 (PDB ID: 1YCR), ii) estrogen receptor β in complex with the nuclear receptor coactivator 5 (Ncoa5; PDB ID: 2J7X), iii) the coiled-coil dimerization motif of Geminin (PDB ID: 1T6F), and iv) the coiled-coil dimerization motif of the human ROCK I protein (PDB ID: 3O0Z) using the Biopolymer and Homology module of Insight 2005 (Accelrys, San Diego).

## Additional Information

**How to cite this article**: Randino, R. *et al*. Investigating the Neuroprotective Effects of Turmeric Extract: Structural Interactions of *β*-Amyloid Peptide with Single Curcuminoids. *Sci. Rep.* **6**, 38846; doi: 10.1038/srep38846 (2016).

**Publisher's note:** Springer Nature remains neutral with regard to jurisdictional claims in published maps and institutional affiliations.

## Supplementary Material

Supplementary Information

## Figures and Tables

**Table 1:** Calculated occurrence rates (%) of MM and PM7 conformers presenting “folded”, “semi-folded” and “extended” conformation.

Cmp	MM			PM7		
	Folded	Semi-folded	Extended	Folded	Semi-folded	Extended
CUR (diketone)	22.0	27.5	50.5	65.5	19.5	15
CUR (keto-enol)	0.0	0.0	100	0.0	5.0	95
CYC	0.0	100	0.0	0.0	100	0.0
THC (diketone)	39.5	47.0	13.5	56.0	35.0	9.0
THC (keto-enol)	16.0	59.5	24.5	37.5	45.0	17.5
