# Supplementary material for: Investigating the Neuroprotective Effects of Turmeric Extract: Structural Interactions of β-Amyloid Peptide with Single Curcuminoids
Source: Sci Rep. 2016 Dec 22;6:38846. doi: 10.1038/srep38846 (PMC5177957; doi:10.1038/srep38846)
Supplement: Supplementary Information [file srep38846-s1.doc]

*SUPPORTING INFORMATION FOR*

**Investigating the Neuroprotective Effects of Turmeric Extract: Structural Interactions of β-Amyloid Peptide with Single Curcuminoids**

Rosario Randino,^1§^ Manuela Grimaldi,^1§^ Marco Persico,^2§^ Augusta De Santis,^3^ Elena Cini, ^4^ Walter Cabri,^5,6^ Antonella Riva,^5^  Gerardino D’Errico,^2^ Caterina Fattorusso,^2*^ Anna Maria D’Ursi,^1*^ Manuela Rodriquez^1*^

*^1^ Department of Pharmacy, University of Salerno, Via Giovanni Paolo II, 132, 84084-Fisciano-Italy*

*^2^ Department of Pharmacy, University of Naples “Federico II”, Via D. Montesano, 49, 80131-Naples-Italy*

*^3^ Department of Chemical Sciences, University of Naples “Federico II”, Via Cinthia, 80126-Naples-Italy*

*^4^ Department of Biotechnology, Chemistry and Pharmacy, University of Siena, Via Aldo Moro, 2, 53100-Siena-Italy*

*^5^ R&D Department, Indena, Viale Ortles, 12, 20139-Milan-Italy*

*^6^ Innovation & Development Fresenius-Kabi, Piazza Maestri del Lavoro, 7, 20063-Cernusco sul Naviglio Milan-Italy*

^*^To whom correspondence may be addressed: [*cfattoru@unina.it*](mailto:cfattoru@unina.it)*,* [*dursi@unisa.it*](mailto:dursi@unisa.it) *and* [*mrodriquez@unisa.it*](mailto:mrodriquez@unisa.it)

^§^These authors contributed equally to this work

**Table of Contents:**

Title, Affiliations, Table of Contents.................................................................................**S1**

Copies of chromatograms of curcuminoids (Figure 1SI; Figure 2SI)………..…….....**S2-S3**

Reaction conditions for cyclocurcumin synthesis (Table 1SI)………..…….....................**S4**

Copies ^1^H and ^13^C NMR of compounds **1-8**.............................................................**S5**-**S18**

EPR studies (Table 2SI)................................................................................................**S19**

Molecular docking studies (Figures 3-11SI; Tables 3-8SI)....................................**S20**-**S34**


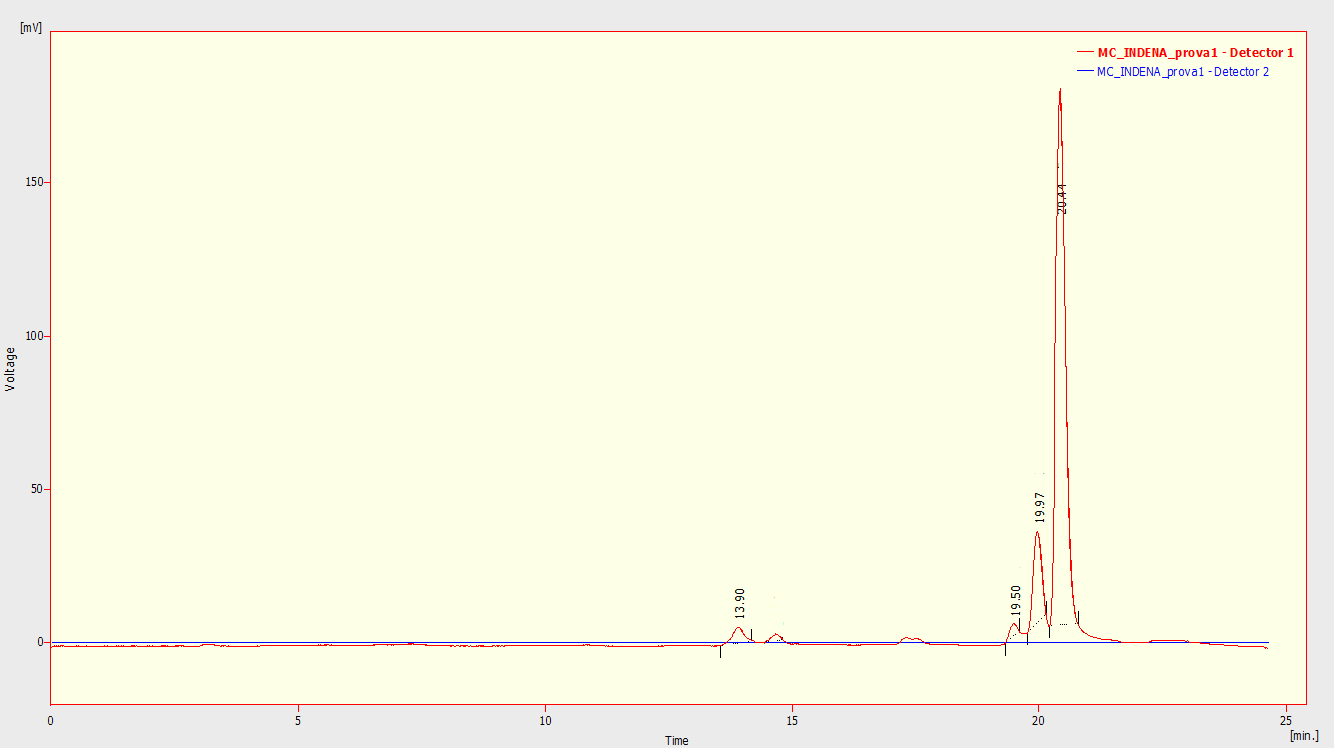


**1**, CUR

**2,** DMC

**3,** BDMC

**4,** CYC

**Figure 1SI.** HPLC chromatogram showing composition of *Curcuma longa* extract as it is in MIX and Meriva® extract.


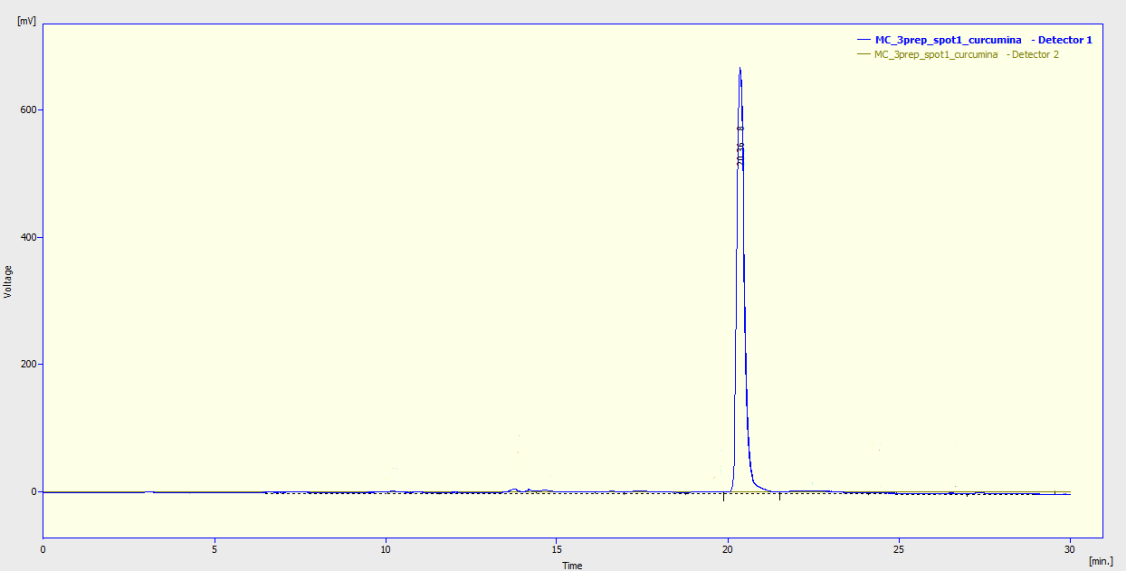

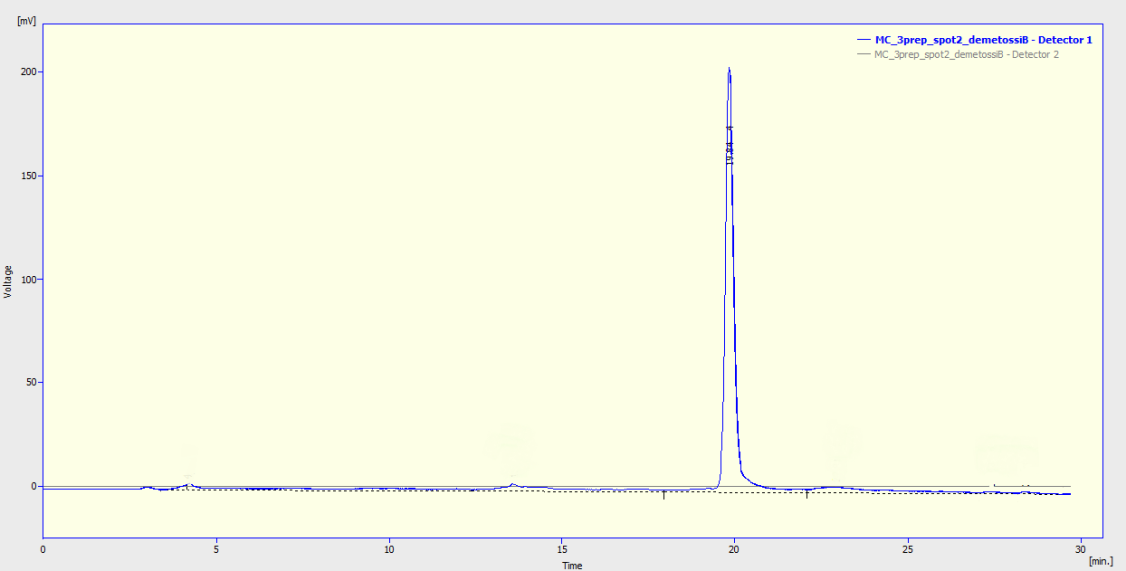

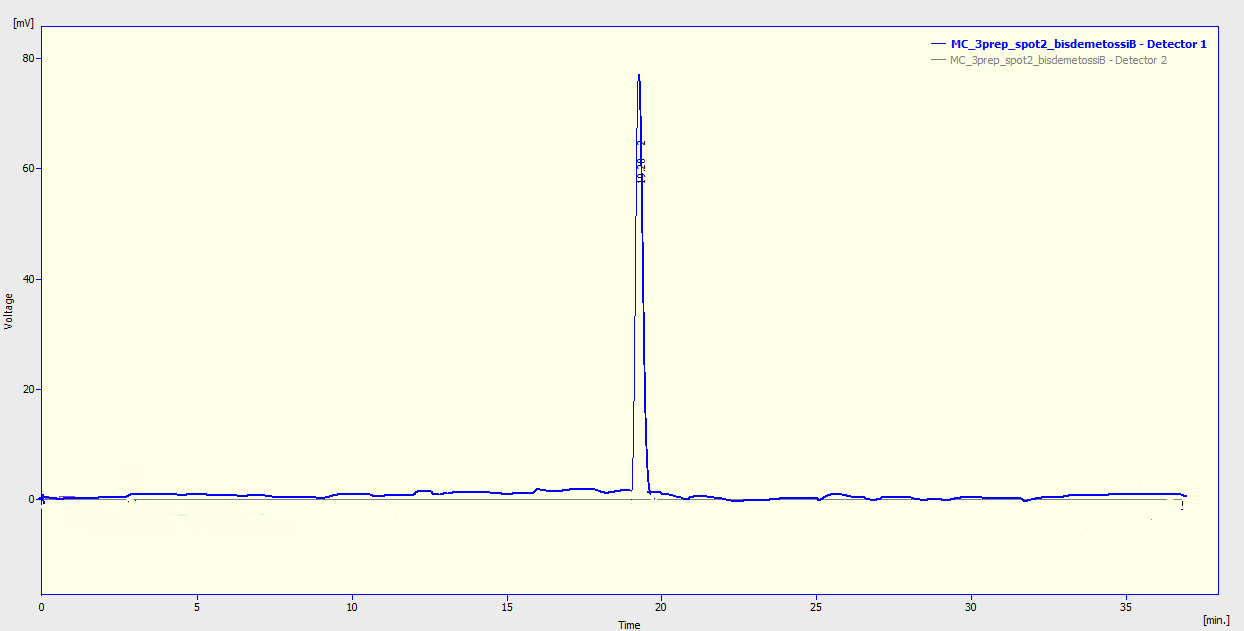

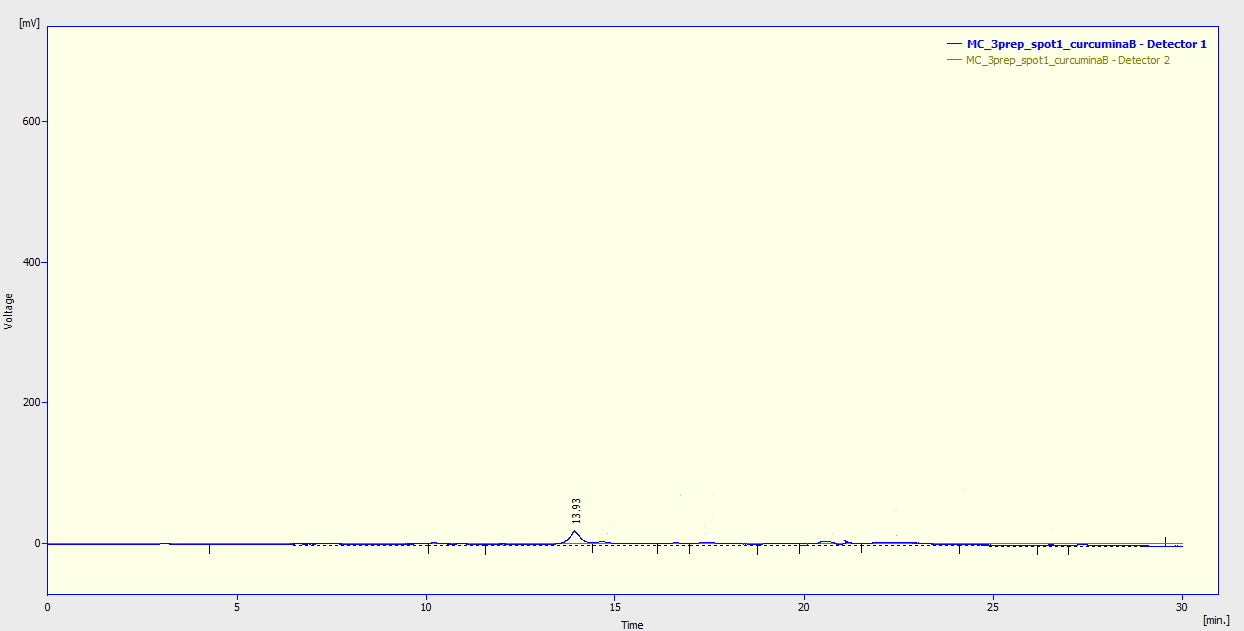


**Figure 2SI**. HPLC Chromatograms *of isolated CUR, DMC, BDMC and CYC.*

**Table 1SI**. Reaction conditions for cyclocurcumin synthesis.

| **Entry** | **Solvent^a^** | **Acid (%)** | **(reaction time, temperature)** | **Yield^b^** |
| --- | --- | --- | --- | --- |
| **1** | Benzene | TFA, 3% | 65 h, rt, dark | 5% |
| **2** | Benzene | TfOH, 3% | 65 h, rt, dark | 5% |
| **MW Conditions (temperature, reaction time)** | | | | |
| **3** | Benzene | TFA, 3% | 100 °C, 4x20 min, | 5% |
| **4** | - | TFA, 100% | 100 °C, 4 min, | **10%** |

^a^ Reaction was carried out in a [0.25 M] benzene solution. ^b^ Yields are evaluated on isolated product.

*1,* ***Curcumin (MeOD)***


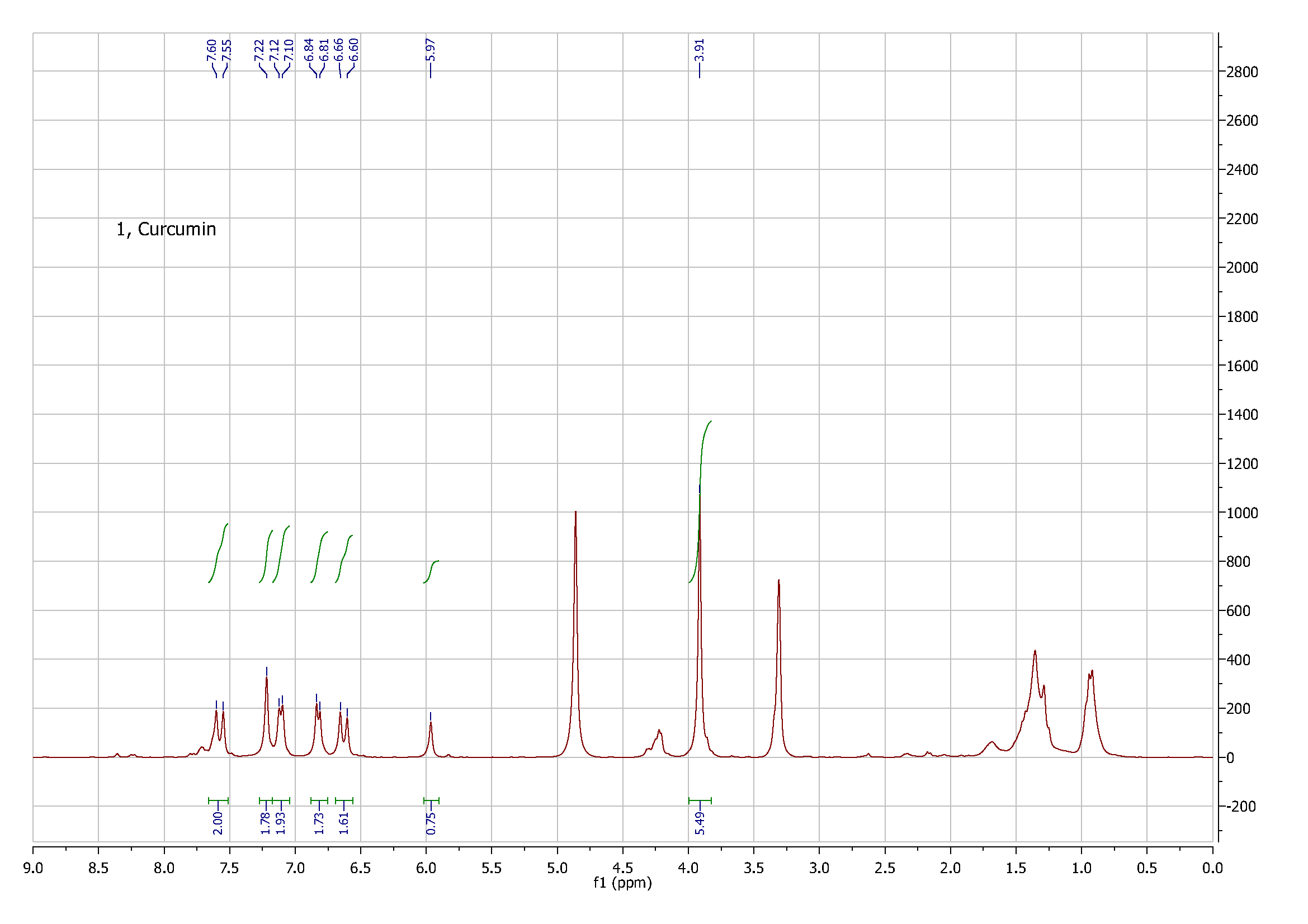


***
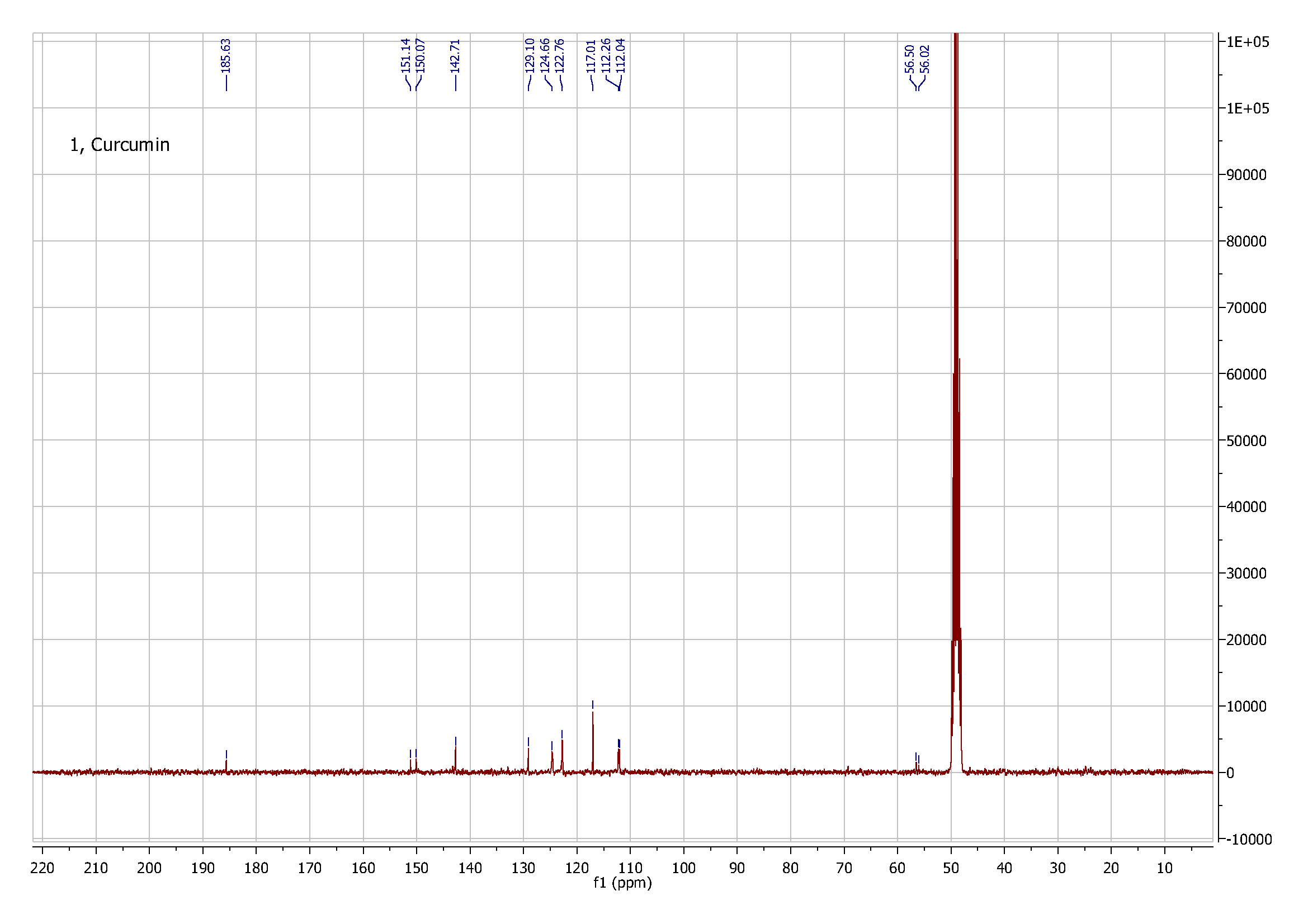
***

***2, Demethoxycurcumin (MeOD)***

***
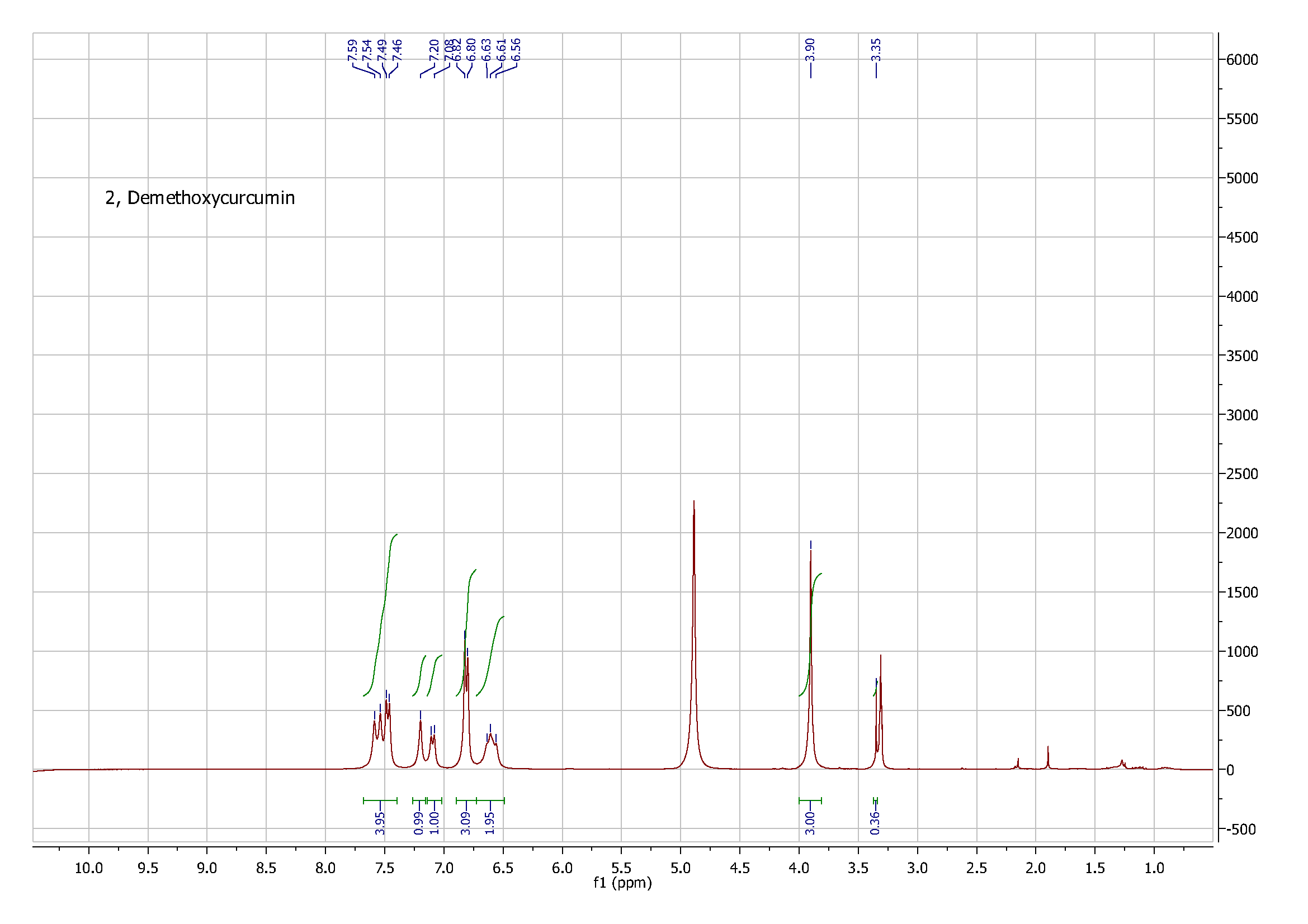
***

***
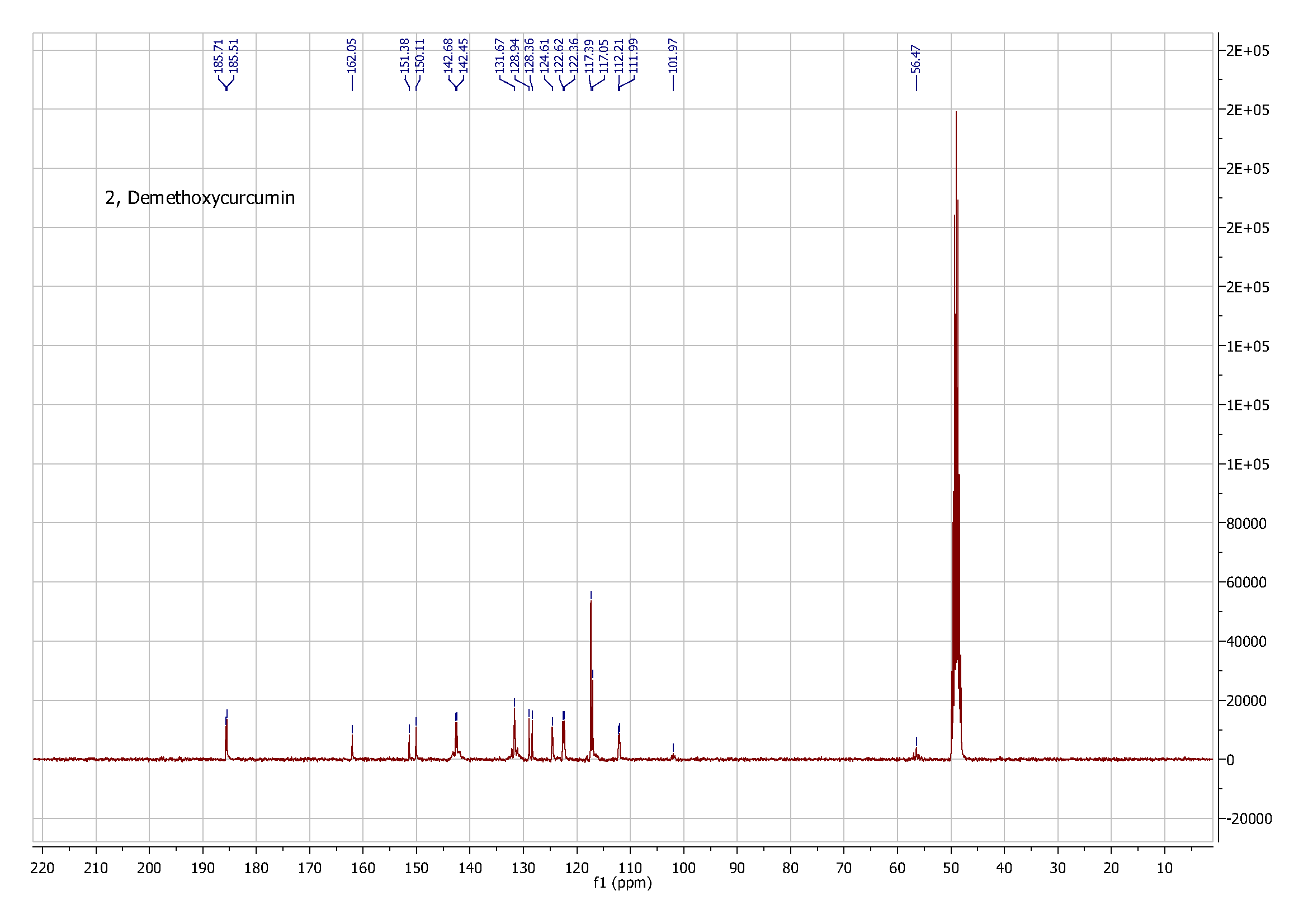
***

***3, Bisdemethoxycurcumin (MeOD)***

***
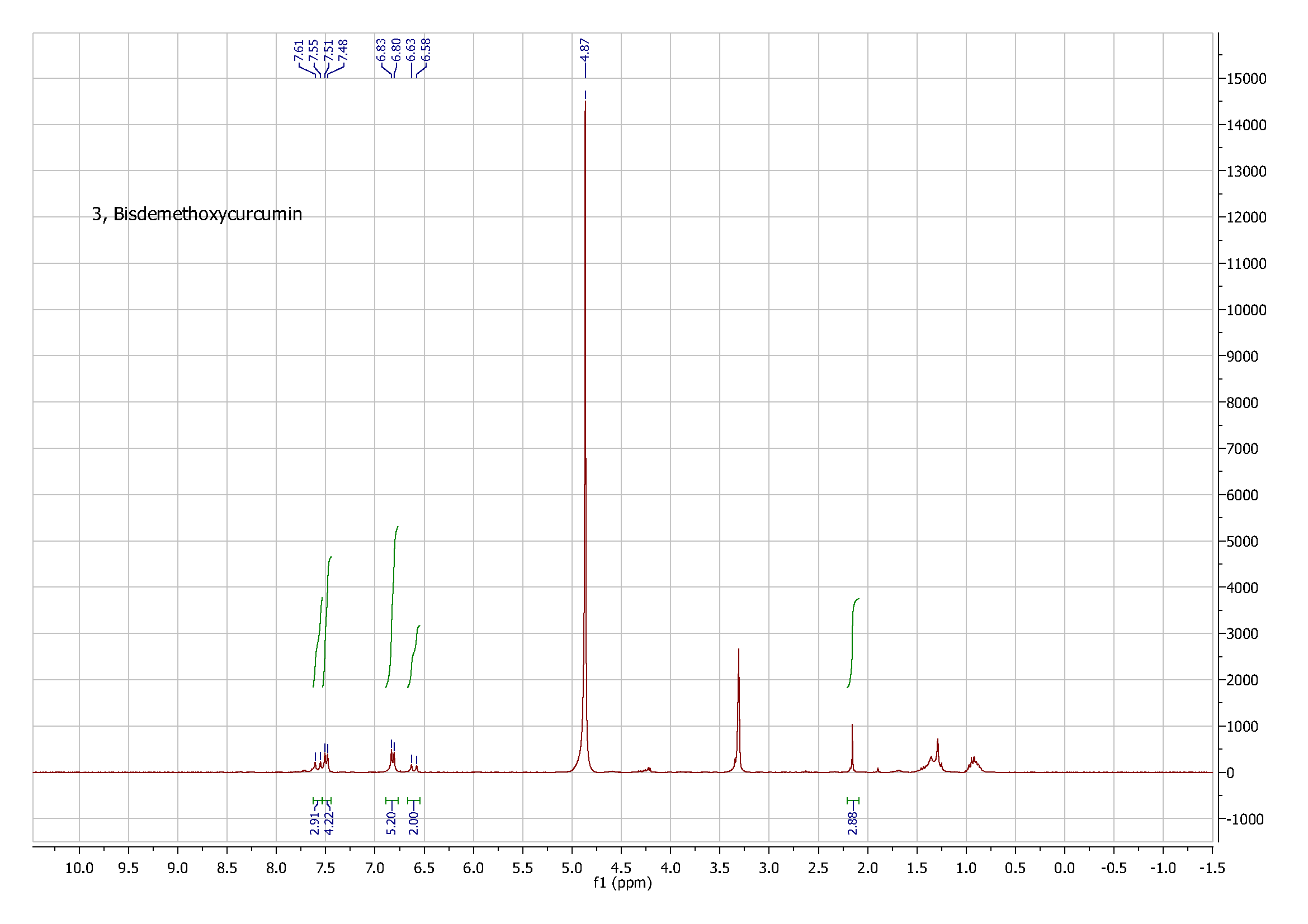
***

***
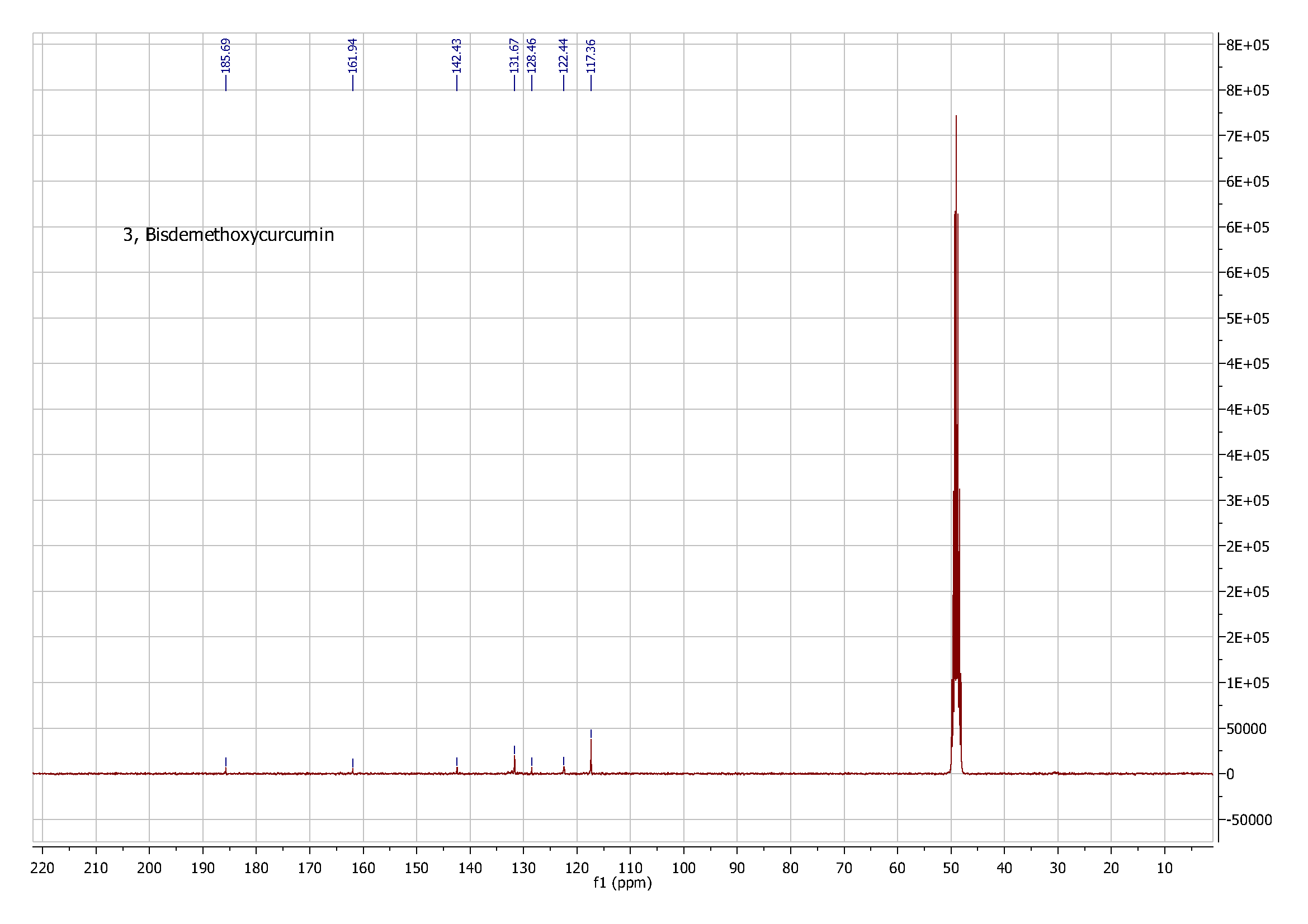
***

***4, Cyclocurcumin (MeOD)***

***
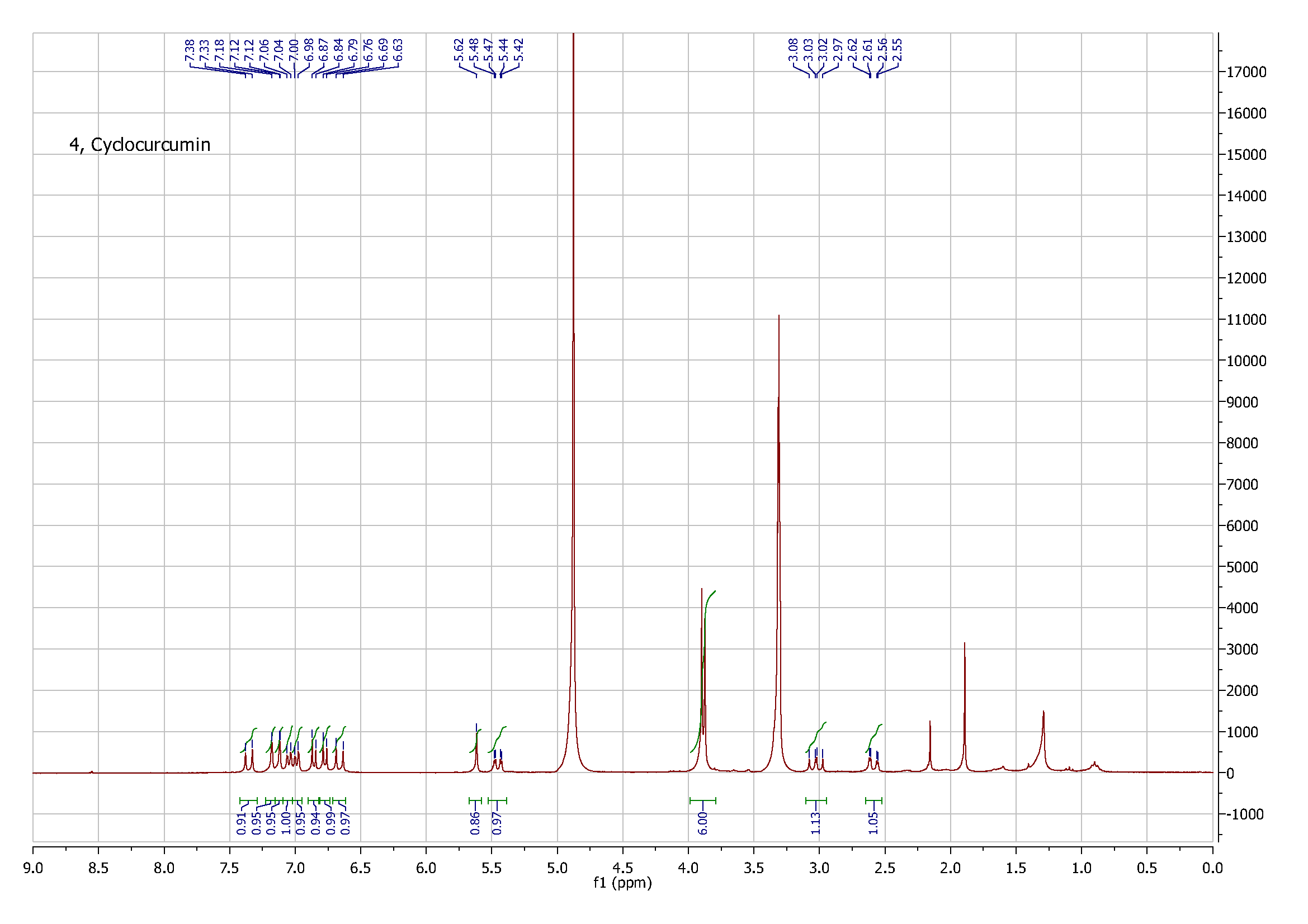
***

***
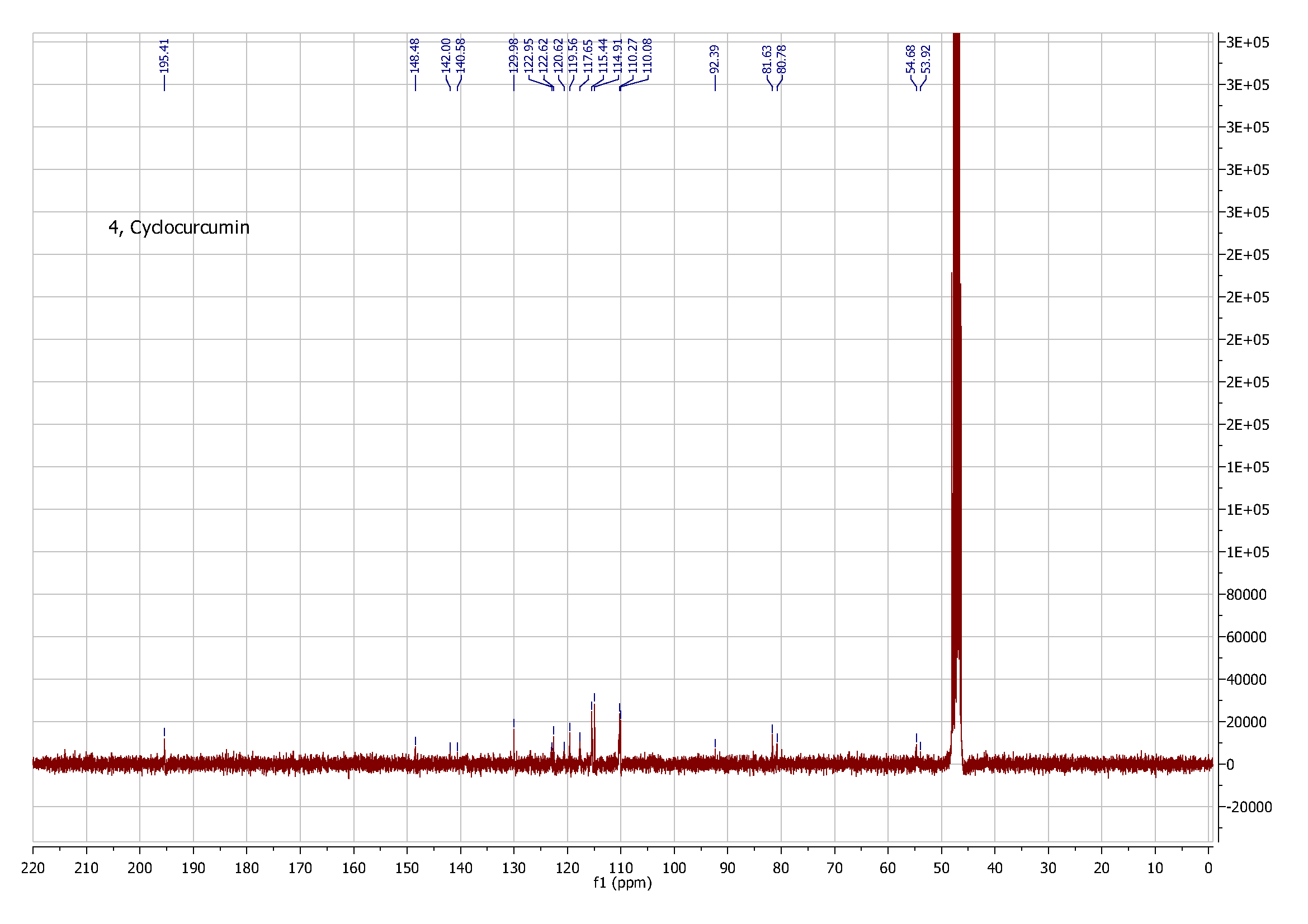
***

***5, Tetrahydrocurcumin (MeOD)***

***
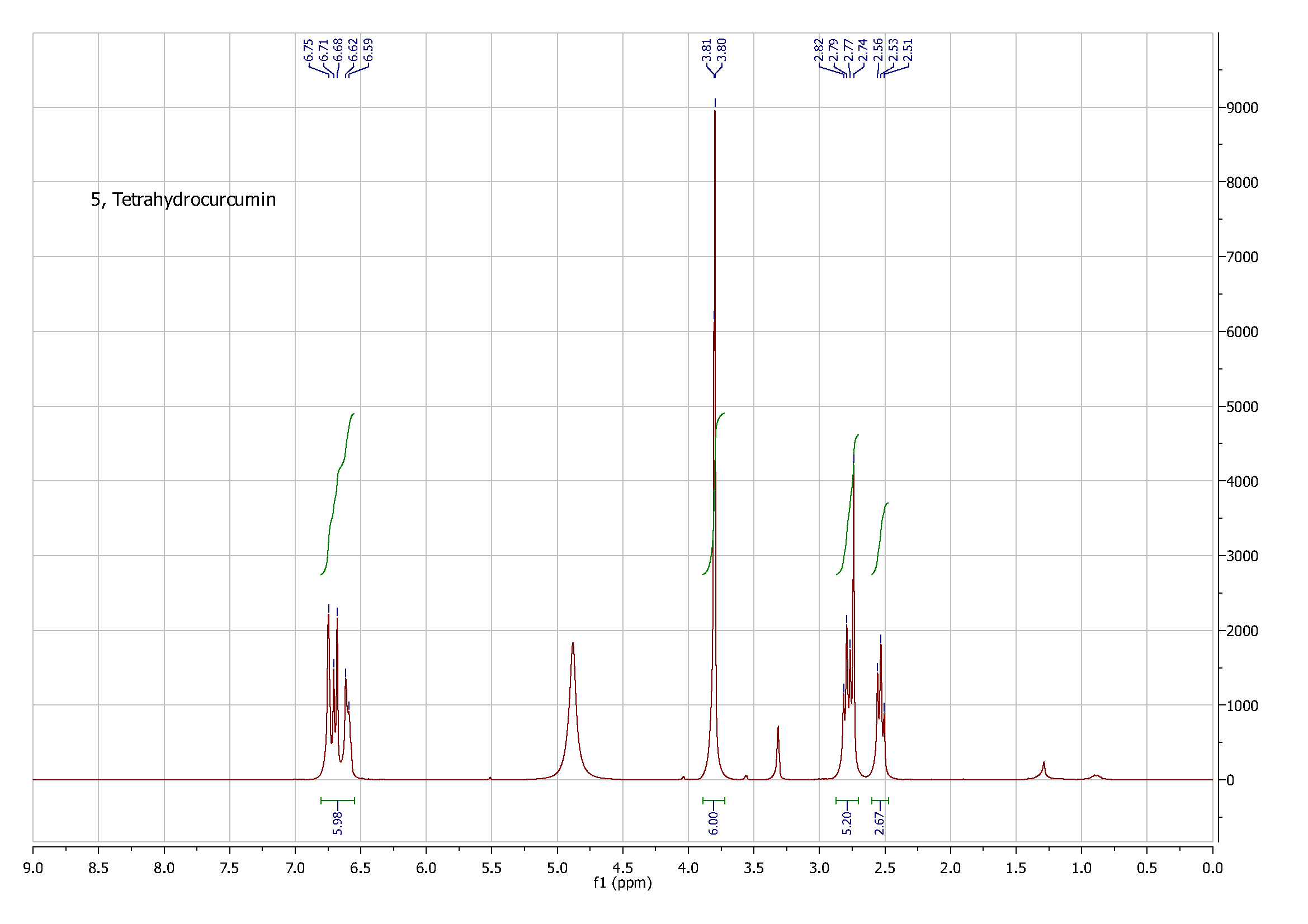
***

***
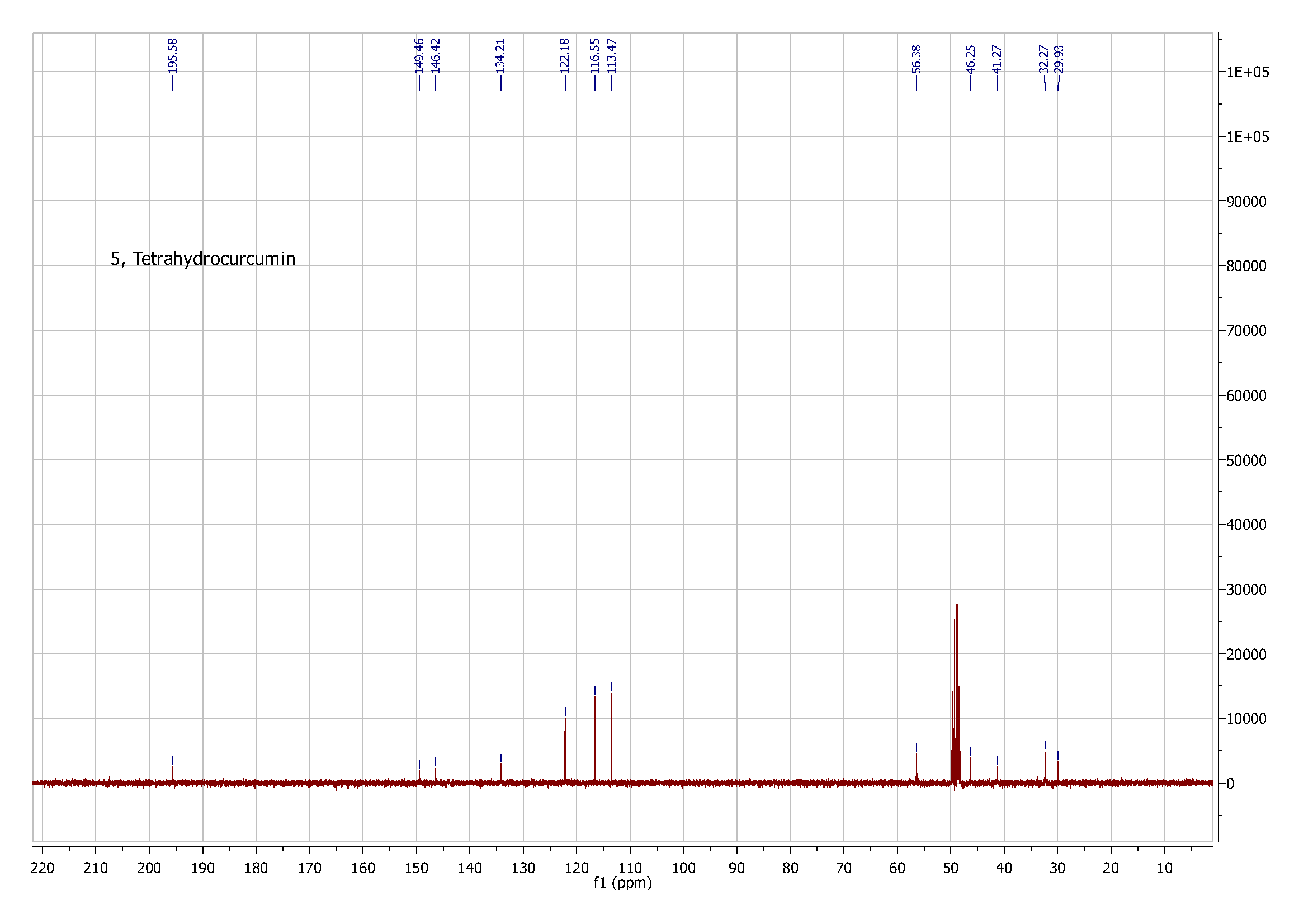
***

***6, Hexahydrocurcumin (MeOD)***

***
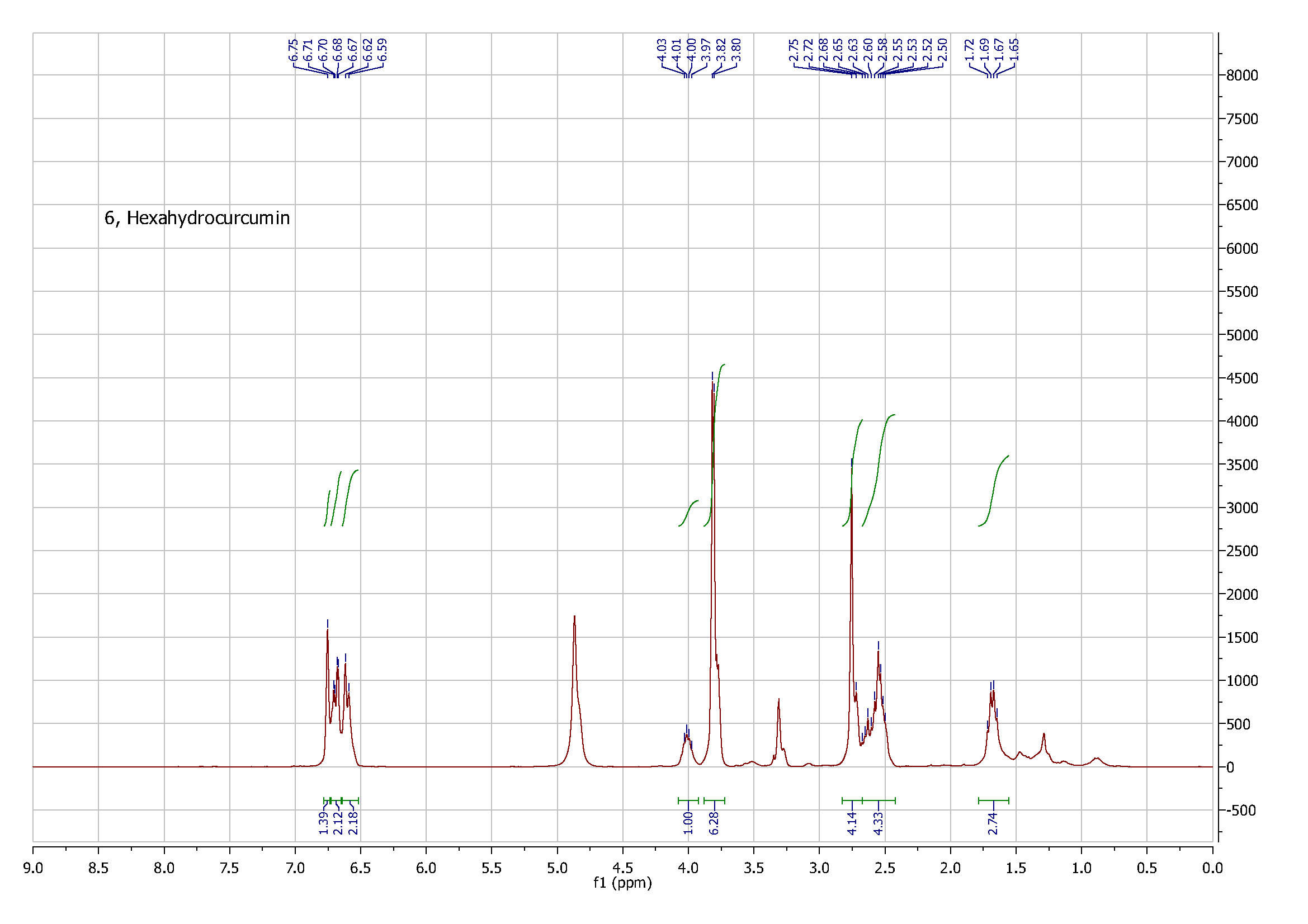
***

***
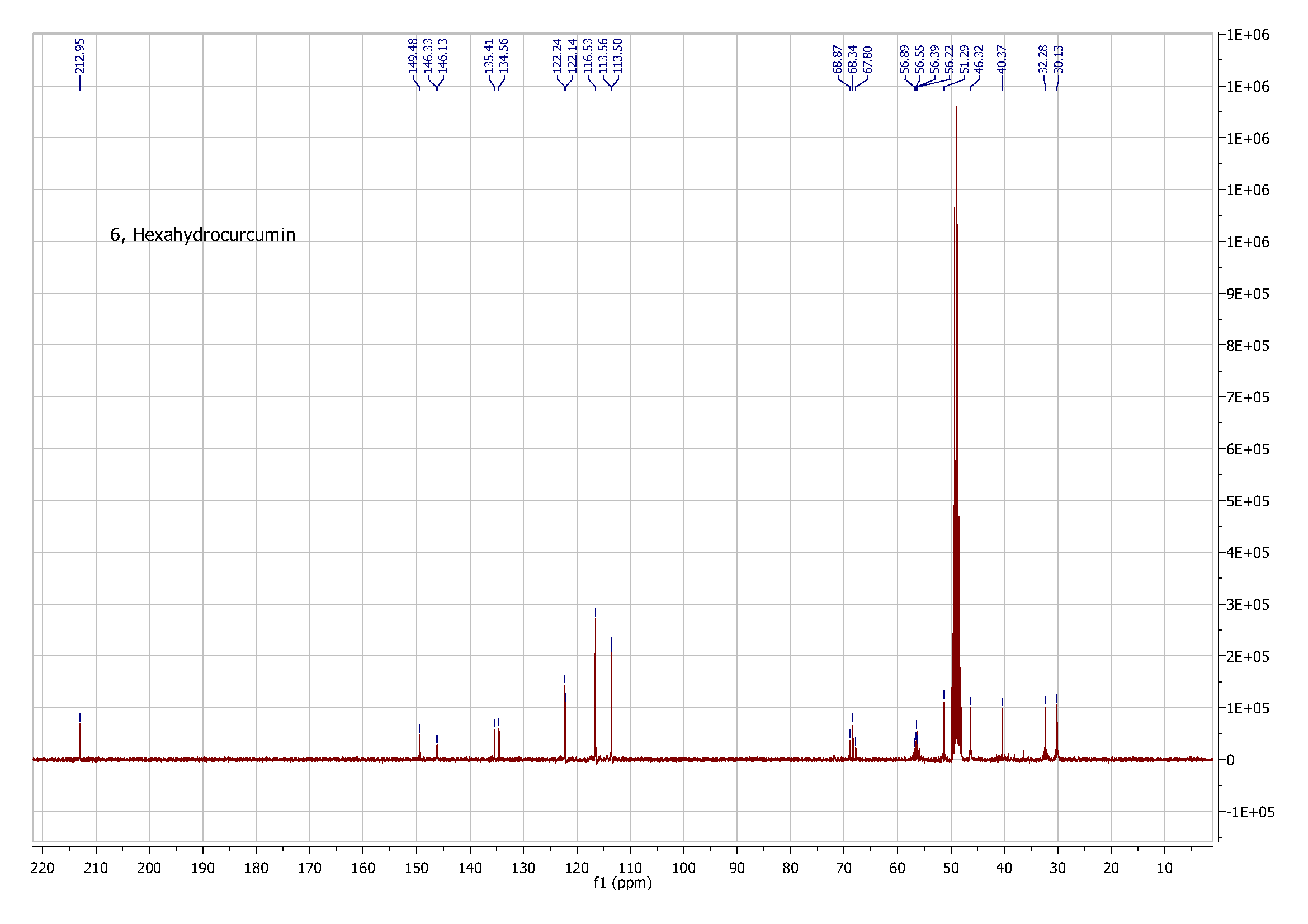
***

***7, Octahydrocurcumin (MeOD)***

***
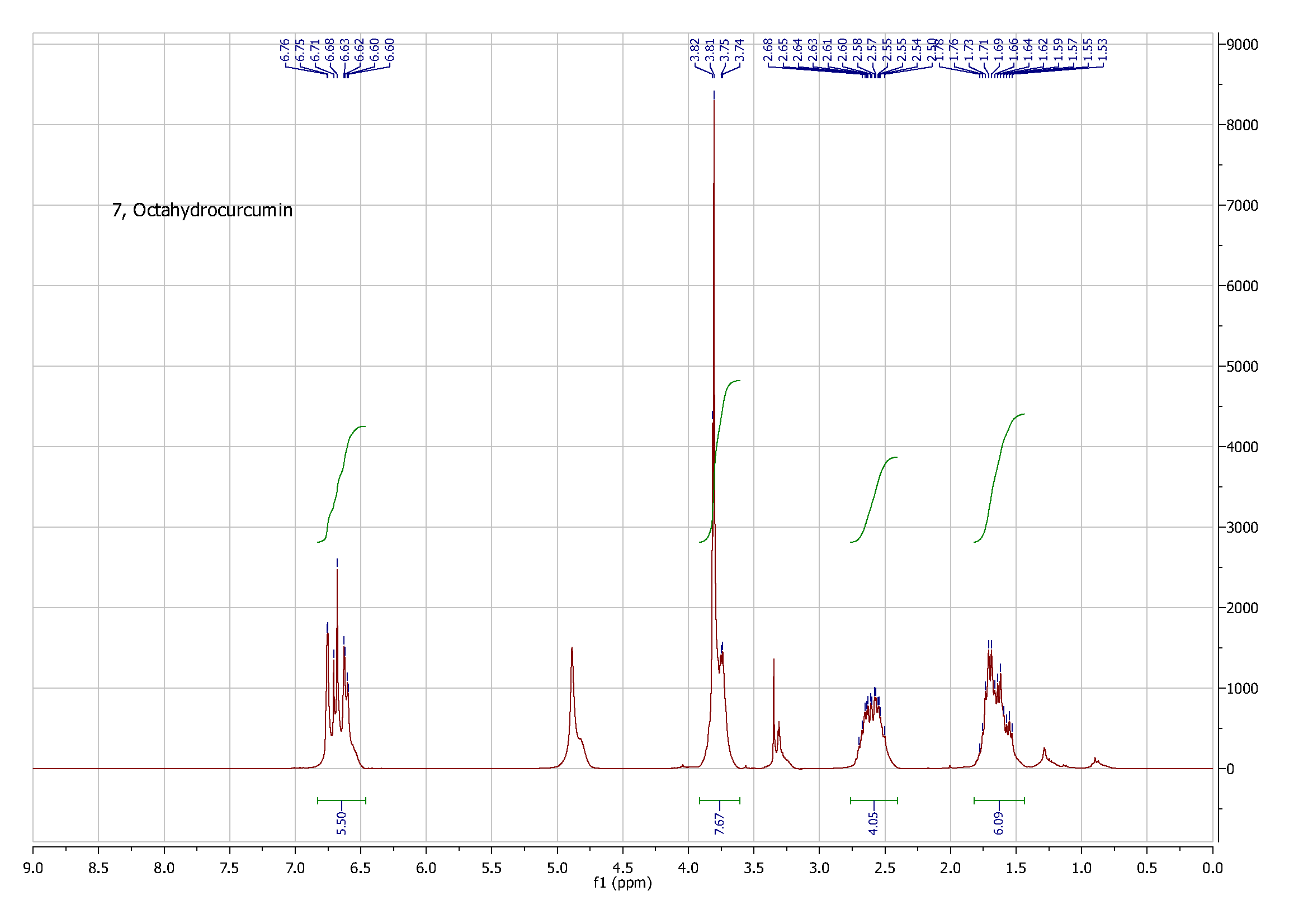
***

***
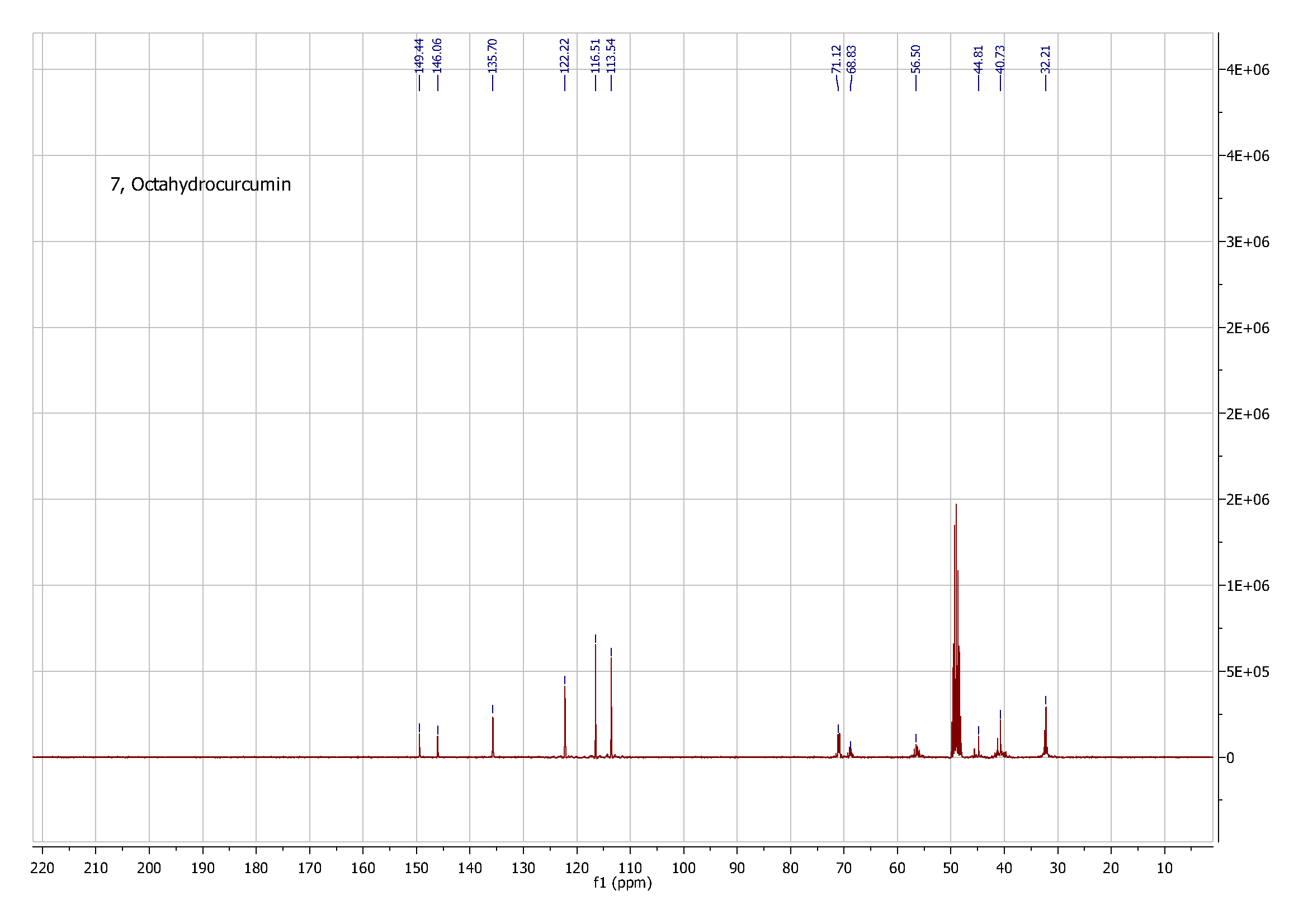
***

***Table 2SI.*** Outer hyperfine splittings (2A_max_) of spin-labeled phospholipids incorporated in DOPC, DOPG, DOPC/DOPG 90/10 lipid bilayers, in the presence of ligands (CUR, DMC, BDMC, MIX, CYC, THC, HHC and OHC) and/or A*β*(25-35).

|  | ***DOPC*** | | ***DOPG*** | | ***DOPC/DOPG 90/10*** | |
| --- | --- | --- | --- | --- | --- | --- |
|  | ***5-PCSL*** | ***14-PCSL*** | ***5-PCSL*** | ***14-PCSL*** | ***5-PCSL*** | ***14-PCSL*** |
|  | *51.5* | *32.2* | *51.2* | *31.7* | *51.3* | *32.6* |
| ***+ CUR*** | *51.9* | *31.9* | *52.6* | *31.8* | *52.0* | *32.8* |
| ***+ DMC*** | *51.8* | *31.8* | *52.7* | *31.7* | *52.1* | *32.8* |
| ***+ BDMC*** | *52.0* | *32.0* | *52.6* | *31.5* | *51.9* | *32.5* |
| ***+ MIX*** | *51.9* | *31.9* | *52.8* | *31.9* | *52.0* | *32.9* |
| ***+ CYC*** | *52.3* | *32.3* | *53.2* | *32.1* | *52.4* | *33.1* |
| ***+ THC*** | *52.1* | *32.2* | *53.0* | *32.0* | *52.2* | *33.0* |
| ***+ HHC*** | *51.9* | *32.0* | *52.8* | *31.6* | *52.0* | *32.8* |
| ***+ OHC*** | *52.0* | *31.9* | *52.9* | *31.9* | *51.9* | *32.5* |

***+Aβ (25-35)***

|  | ***DOPC*** | | ***DOPG*** | | ***DOPC/DOPG 90/10*** | |
| --- | --- | --- | --- | --- | --- | --- |
|  | ***5-PCSL*** | ***14-PCSL*** | ***5-PCSL*** | ***14-PCSL*** | ***5-PCSL*** | ***14-PCSL*** |
| ***Aβ (25-35)*** | *52.2* | *32.0* | *52.0* | *31.4* | *52.1* | *32.5* |
| ***+ CUR*** | *52.0* | *31.7* | *52.8* | *31.6* | *52.1* | *32.9* |
| ***+ DMC*** | *51.8* | *31.9* | *52.8* | *31.7* | *52.2* | *32.9* |
| ***+ BDMC*** | *52.1* | *32.0* | *52.6* | *31.8* | *51.9* | *32.8* |
| ***+ MIX*** | *51.8* | *31.7* | *52.7* | *31.6* | *52.1* | *32.7* |
| ***+ CYC*** | *52.5* | *32.1* | *53.6* | *32.0* | *52.5* | *32.9* |
| ***+ THC*** | *52.3* | *32.0* | *53.2* | *32.0* | *52.4* | *32.8* |
| ***+ HHC*** | *51.9* | *31.8* | *52.8* | *31.9* | *52.3* | *32.7* |
| ***+ OHC*** | *51.9* | *31.9* | *53.0* | *31.9* | *52.1* | *32.8* |

**Figure 3SI.** Obtained PM7 (orange) and MM (blue) conformers of di-ketone CUR plotted against the distance between the centroids of the two aromatic ring (d1).

***
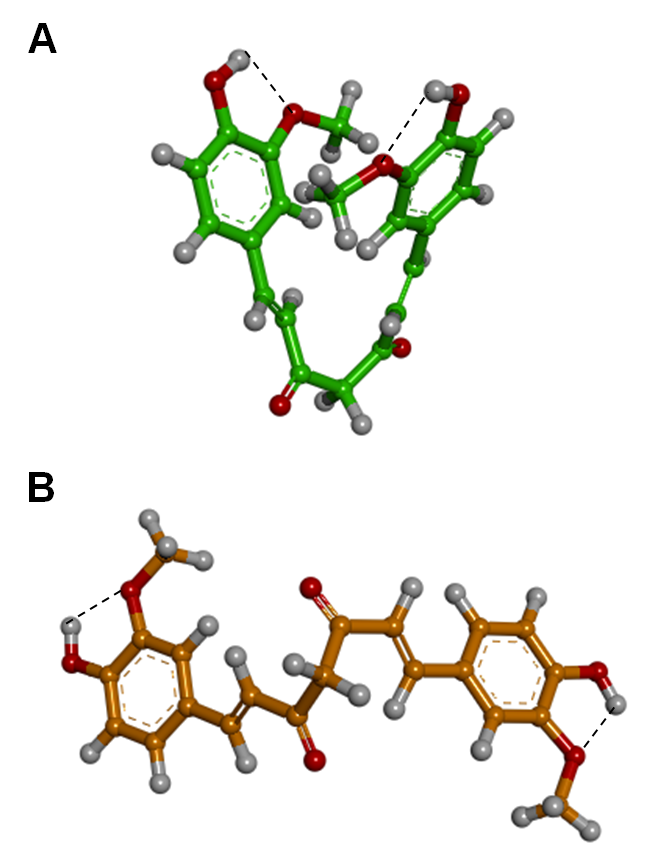
***

**Figure 4SI.** PM7 lowest energy conformers of di-ketone CUR: “folded” (A; green) and “extended” (B; orange) conformational family. The conformers are displayed as ball&sticks and colored by atom type: O, red; H, white. Hydrogen bonds are highlighted with a black dashed line.

**Figure 5SI.** Obtained PM7 (orange) and MM (blue) conformers of keto-enol CUR plotted against the distance between the centroids of the two aromatic rings (d1).

**Figure 6SI.** Obtained PM7 (orange) and MM (blue) conformers of CYC plotted against the distance between the centroids of the two aromatic rings (d1).

**Figure 7SI.** Obtained PM7 (orange) and MM (blue) conformers of di-ketone THC plotted against the distance between the centroids of the two aromatic rings (d1).

**Figure 8SI.** Obtained PM7 (orange) and MM (blue) conformers of keto-enol THC plotted against the distance between the centroids of the two aromatic rings (d1).

***
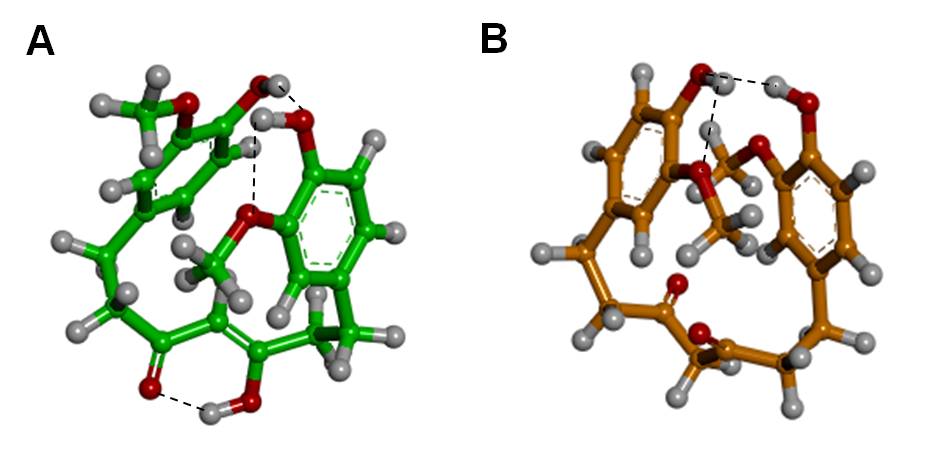
***

**Figure 9SI.** PM7 lowest energy conformers of THC in keto-enol (A; green) and diketone form (B; orange). The conformers are displayed as ball&sticks and colored by atom type: O, red; H, white. Hydrogen bonds are highlighted with a black dashed line.


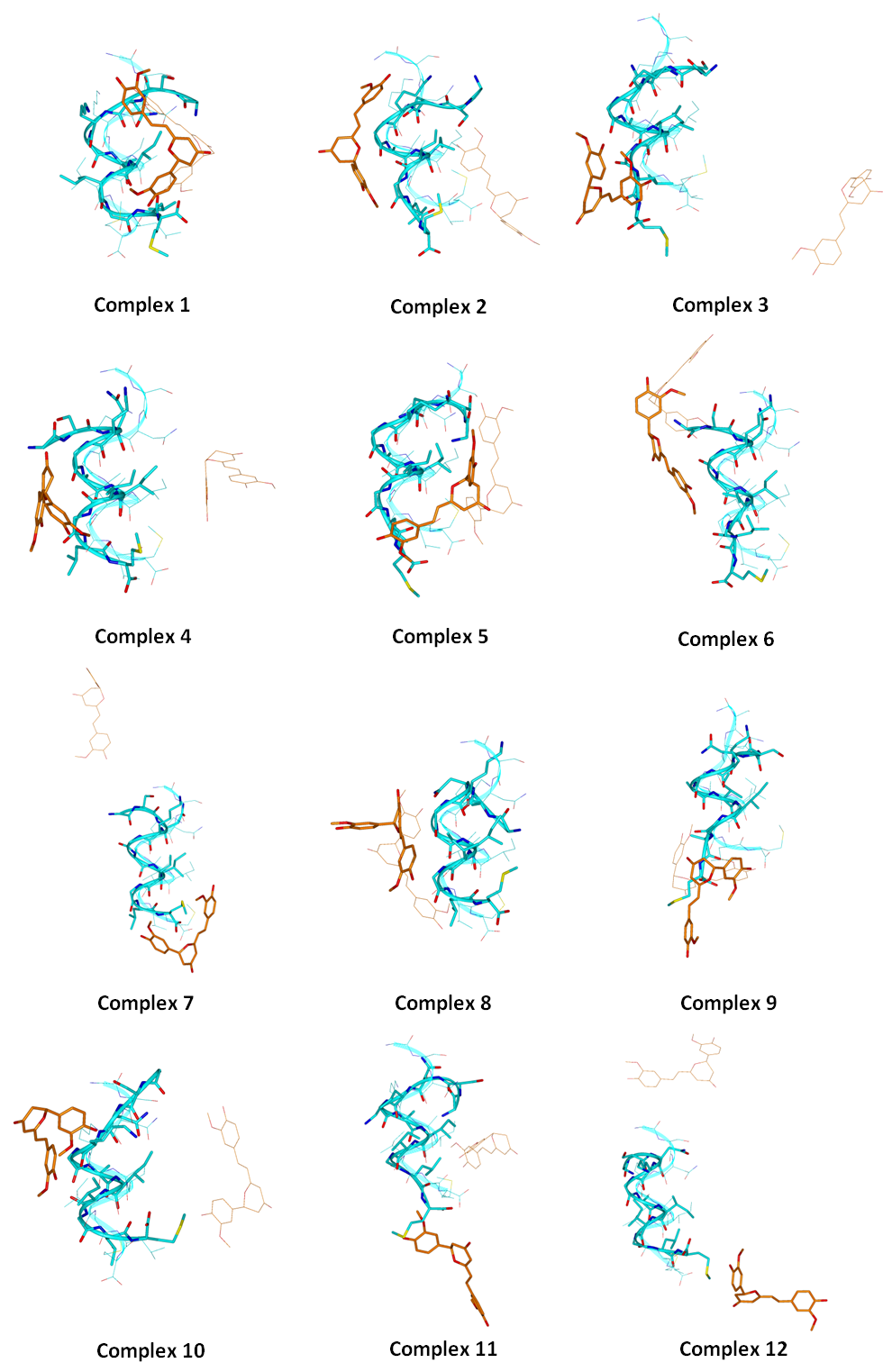


**Figure 10SI.** Superimposition between docked (lines) and annealed complexes (sticks) of CYC (orange) and Aβ(25–35) peptide (cyan). The peptides are displayed as ribbon. Molecules are colored by atom type (O: red; N: blue; S: yellow). Hydrogens are omitted for sake of clarity.


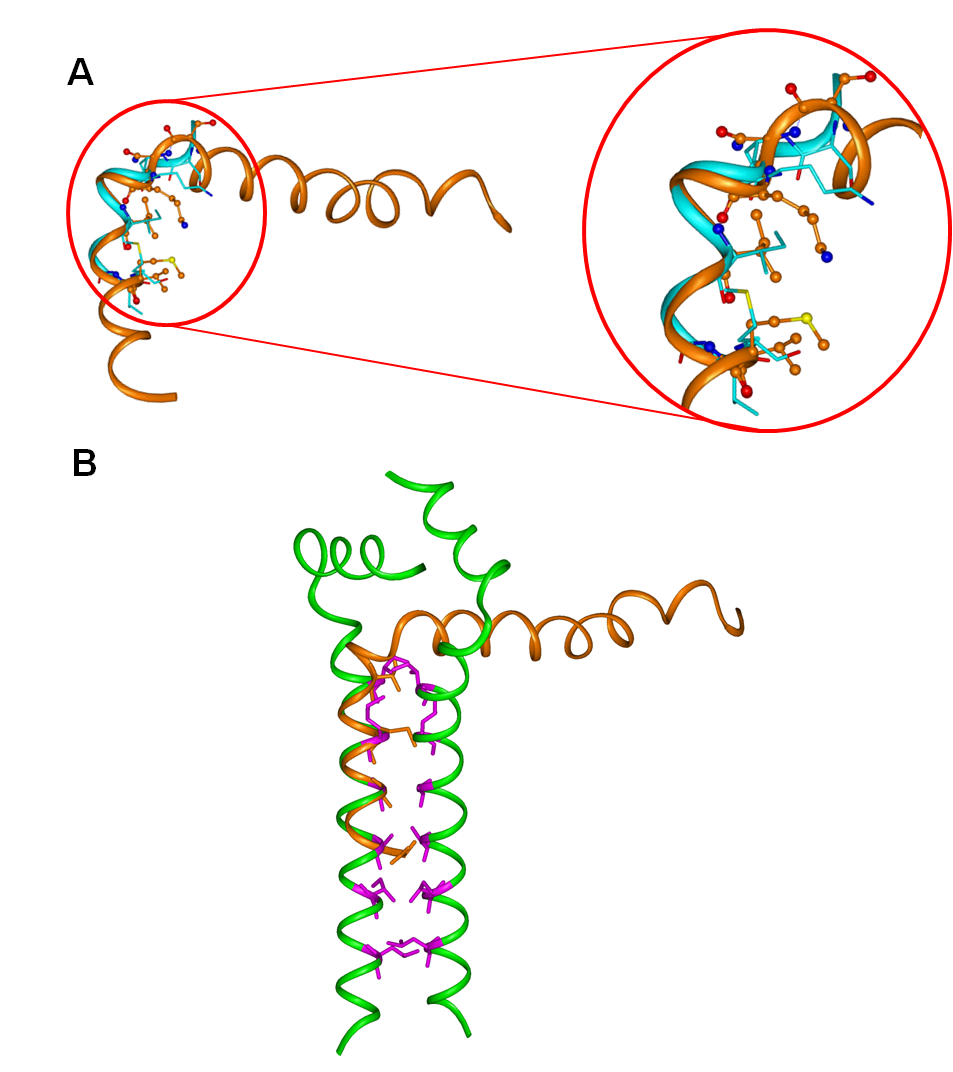


**Figure 11SI.** A) Superimposition between Aβ(25–35) (cyan and sticks; PDB ID: 1QWP) and Aβ(1–42) peptide (orange and ball&sticks; PDB ID: 1IYT). The peptides are displayed as ribbon and colored by atom type (O: red; N: blue; S: yellow). B) Superimposition between Aβ(1–42) peptide (orange ; PDB ID: 1IYT) and APP (green; PDB ID: 2LOH). The heptad repeat motif interacting amino acids are displayed as stick and in case of APP colored in magenta. Hydrogens are omitted for sake of clarity.

**Table 3SI.** ΔE range values (kcal/mol), distance X-Y (d1), torsional angle values (τ1, τ2, τ3, and τ4), and occurrence rate (%) of each conformational family of CUR in diketone form considering MM and PM7 conformers within 5 kcal/mol from the global minimum.

| **Fam** | **ΔE_GM_ (kcal/mol)^a^** | **d1 (Å)^a^** | **Torsional Angles (°)^a^** | | | | **%** |
| --- | --- | --- | --- | --- | --- | --- | --- |
|  |  |  | **τ1^b^** | **τ2^c^** | **τ3^d^** | **τ4^e^** |  |
| **MM I** | 0.00 | 5.10 | -128.37 | 78.66 | -74.92 | 134.23 | 23 |
| **MM II** | 0.01 | 5.10 | 134.50 | -74.85 | 78.67 | -128.26 | 29 |
| **MM III** | 2.56 | 5.61 | -143.50 | 38.42 | 38.48 | -143.52 | 3 |
| **MM IV** | 3.17 | 4.87 | 134.07 | -35.71 | -35.53 | 133.88 | 1 |
| **MM V** | 3.76 | 11.35 | 178.37 | 86.74 | 86.73 | 178.36 | 22 |
| **MM VI** | 3.76 | 11.35 | -178.39 | -86.75 | -86.74 | -178.36 | 22 |
| **PM7 I** | 0.00 | 4.98 | -153.51 | 32.16 | 32.09 | -153.48 | 26 |
| **PM7 II** | 0.00 | 4.98 | 153.49 | -32.13 | -32.13 | 153.50 | 23 |
| **PM7 III** | 0.95 | 4.52 | 154.65 | -71.99 | 52.78 | -117.36 | 24 |
| **PM7 IV** | 0.95 | 4.52 | -154.57 | 71.95 | -52.85 | 117.48 | 24 |
| **PM7 V** | 1.45 | 4.64 | 166.86 | -158.96 | 17.27 | -75.06 | 1 |
| **PM7 VI** | 2.88 | 3.68 | -100.07 | -31.34 | 61.42 | -152.66 | 1 |
| **PM7 VII** | 3.42 | 3.78 | 153.25 | -49.10 | -0.93 | 130.31 | 1 |

*^a^The values reported refer to the lowest energy conformers of the family. ^b^τ1 torsional angle is calculated considering a, b, c, and d atoms. ^c^τ2 torsional angle is calculated considering b, c, d, and e atoms. ^d^τ3 torsional angle is calculated considering c, d, e, and f atoms. ^e^τ4 torsional angle is calculated considering d, e, f, and g atoms.*

**Table 4SI.** ΔE range values (kcal/mol), distance X-Y (d1), torsional angle values (τ1, τ2, τ3, and τ4), and occurrence rate (%) of each conformational family of CUR in keto-enol form considering MM and PM7 conformers within 5 kcal/mol from the global minimum.

| **Fam** | **ΔE_GM_ (kcal/mol)^a^** | **d1 (Å)^a^** | **Torsional Angles (°)^a^** | | | | **%** |
| --- | --- | --- | --- | --- | --- | --- | --- |
|  |  |  | **τ1^b^** | **τ2^c^** | **τ3^d^** | **τ4^e^** |  |
| **MM I** | 0.00 | 12.31 | 178.92 | -179.95 | -180.00 | 179.90 | 33 |
| **MM II** | 1.05 | 11.58 | 29.55 | -174.12 | 179.78 | -179.84 | 39 |
| **MM III** | 3.41 | 11.54 | -174.17 | 42.94 | 179.33 | 179.80 | 18 |
| **MM IV** | 4.25 | 10.54 | 21.65 | 48.31 | 179.53 | 179.64 | 10 |
| **PM7 I** | 0.00 | 12.45 | 177.66 | 179.69 | 179.84 | 176.22 | 42.5 |
| **PM7 II** | 0.81 | 11.63 | -42.74 | 179.36 | 179.71 | 177.96 | 57.5 |

*^a^ The values reported refer to the lowest energy conformers of the family. ^b^τ1 torsional angle is calculated considering a, b, c, and d atoms. ^c^ τ2 torsional angle is calculated considering b, c, d, and e atoms. ^d^ τ3 torsional angle is calculated considering c, d, e, and f atoms. ^e^ τ4 torsional angle is calculated considering d, e, f, and g atoms.*

**Table 5SI.** ΔE range values (kcal/mol), distance X-Y (d1), torsional angle values (τ1, τ2, and τ3), and occurrence rate (%) of each conformational family of CYC considering MM and PM7 conformers within 5 kcal/mol from the global minimum.

| **Fam** | **ΔE_GM_ (kcal/mol)^a^** | **d1 (Å)^a^** | **Torsional Angles (°)^a^** | | | **%** |
| --- | --- | --- | --- | --- | --- | --- |
|  |  |  | **τ1^b^** | **τ2^c^** | **τ3^d^** |  |
| **MM I** | 0.00 | 8.81 | 179.31 | 46.79 | -48.30 | 23 |
| **MM II** | 1.89 | 8.37 | 179.35 | -41.19 | 37.12 | 11 |
| **MM III** | 2.53 | 9.66 | -0.61 | 46.81 | -49.20 | 51 |
| **MM IV** | 4.42 | 8.83 | 0.48 | -41.46 | 37.89 | 15 |
| **PM7 I** | 0.00 | 7.00 | 176.89 | -52.12 | 54.91 | 11 |
| **PM7 II** | 0.39 | 7.60 | 31.83 | -51.73 | 50.09 | 28 |
| **PM7 III** | 0.84 | 8.05 | -166.05 | 51.25 | -41.51 | 19 |
| **PM7 IV** | 1.57 | 9.02 | 21.02 | 50.69 | -40.61 | 42 |

*^a^ The values reported refer to the lowest energy conformers of the family. ^b^τ1 torsional angle is calculated considering a, b, c, and d atoms. ^c^τ2 torsional angle is calculated considering e, f, g, and h atoms. ^d^τ3 torsional angle is calculated considering f, g, h, and c atoms.*

**Table 6SI.** ΔE range values (kcal/mol), distance X-Y (d1), torsional angle values (τ1, τ2, τ3, and τ4), and occurrence rate (%) of each conformational family of THC in keto-enol form considering MM and PM7 conformers within 5 kcal/mol from the global minimum.

| **Fam** | **ΔE_GM_ (kcal/mol)^a^** | **d1 (Å)^a^** | **Torsional Angles (°)^a^** | | | | **%** |
| --- | --- | --- | --- | --- | --- | --- | --- |
|  |  |  | **τ1^b^** | **τ2^c^** | **τ3^d^** | **τ4^e^** |  |
| **MM I** | 0.00 | 4.37 | 87.13 | 144.17 | 179.27 | 77.36 | 4 |
| **MM II** | 0.15 | 4.20 | -113.67 | 126.61 | 179.43 | 42.32 | 15 |
| **MM III** | 0.19 | 4.32 | -85.72 | -151.04 | -179.45 | -75.97 | 7 |
| **MM IV** | 0.64 | 4.53 | 98.50 | 157.16 | 178.34 | -105.06 | 27 |
| **MM V** | 1.00 | 4.54 | -112.37 | -71.81 | 179.38 | -105.52 | 8 |
| **MM VI** | 1.68 | 4.66 | -117.31 | 32.08 | 178.13 | -81.03 | 6 |
| **MM VII** | 2.06 | 4.91 | 52.14 | 36.05 | -176.52 | 107.17 | 8 |
| **MM VIII** | 2.16 | 4.67 | 118.45 | -33.51 | -178.08 | 80.42 | 1 |
| **MM IX** | 2.31 | 5.86 | 84.09 | -164.44 | 174.36 | -89.34 | 8 |
| **MM X** | 2.32 | 5.94 | -83.70 | 161.17 | -174.09 | 90.51 | 5 |
| **MM XI** | 3.90 | 5.13 | -60.95 | -32.10 | 174.43 | -84.24 | 3.5 |
| **MM XII** | 3.90 | 5.56 | -108.72 | 55.51 | -172.38 | 83.55 | 1 |
| **MM XIII** | 4.22 | 5.91 | 82.15 | 134.71 | -172.26 | 86.85 | 5 |
| **XIV** | 4.95 | 6.75 | 107.39 | -61.20 | 174.08 | -106.06 | 1 |
| **PM7 I** | 0.00 | 4.03 | 66.27 | 179.47 | 178.72 | 73.41 | 29 |
| **PM7 II** | 0.00 | 4.03 | -66.08 | -179.46 | -178.73 | -73.58 | 42 |
| **PM7 III** | 0.68 | 4.27 | -134.32 | 179.13 | 179.64 | 103.24 | 17 |
| **PM7 IV** | 2.62 | 4.94 | 84.33 | -175.86 | 170.86 | -62.62 | 8 |
| **PM7 V** | 4.95 | 6.03 | 94.46 | -164.28 | 171.53 | -13.47 | 4 |

*^a^ The values reported refer to the lowest energy conformers of the family. ^b^τ1 torsional angle is calculated considering a, b, c, and d atoms. ^c^τ2 torsional angle is calculated considering b, c, d, and e atoms. ^d^τ3 torsional angle is calculated considering c, d, e, and f atoms. ^e^τ4 torsional angle is calculated considering d, e, f, and g atoms.*

**Table 7SI.** Torsional angle values (τ1, τ2, τ3, and τ4) of the experimentally determined structures of CUR.

| **CSD Code** | **Torsional Angles (°)** | | | |
| --- | --- | --- | --- | --- |
|  | **τ1^a^** | **τ2^b^** | **τ3^c^** | **τ4^s^** |
| **AXOGIE** | 176.19 | -177.52 | -178.61 | 179.72 |
| **AXOGOK** | -176.21 | 178.27 | -176.81 | 171.57 |
| **BINMEQ06** | -170.44 | -176.13 | 179.87 | -173.35 |
| **BINMEQ07** | 170.47 | 175.68 | -177.70 | 171.97 |
| **BINMEQ08** | -170.61 | -176.32 | 179.67 | -173.78 |
| **BINMEQ** | 18.39 | 176.43 | -178.81 | -176.99 |
| **BINMEQ01** | -16.63 | -176.17 | 178.93 | 176.88 |
| **BINMEQ02** | 17.31 | 176.18 | -178.91 | -177.12 |
| **BINMEQ03** | -18.32 | -176.71 | 178.83 | 177.74 |
| **BINMEQ04** | -18.49 | -176.41 | 178.83 | 177.07 |
| **BINMEQ05** | 18.60 | 176.31 | -178.84 | -177.05 |

*^a^τ1 torsional angle is calculated considering a, b, c, and d atoms. ^b^τ2 torsional angle is calculated considering b, c, d, and e atoms. ^c^τ3 torsional angle is calculated considering c, d, e, and f atoms. ^d^τ4 torsional angle is calculated considering d, e, f, and g atoms.*

**Table 8SI**. Conformational and interaction energy values of the CYC-Aβ(25–35) peptide complexes obtained by docking studies.

| **Frame** | **Complex Energy**  **(kcal/mol)** | **Non-bond**  **Interaction Energy**  **(kcal/mol)** |
| --- | --- | --- |
| 1 | 173.850 | -17.266 |
| 2 | 177.898 | -14.191 |
| 3 | 180.192 | -14.528 |
| 4 | 182.297 | -14.452 |
| 5 | 185.506 | -12.605 |
| 6 | 187.292 | -12.187 |
| 7 | 187.773 | -12.320 |
| 8 | 188.072 | -10.014 |
| 9 | 188.519 | -9.498 |
| 10 | 189.290 | -13.001 |
| 11 | 193.457 | -5.615 |
| 12 | 198.052 | -2.451 |
